# Sequentiality of beetle communities in the longitudinal gradient of a lowland river in the context of the river continuum concept

**DOI:** 10.7717/peerj.13232

**Published:** 2022-04-05

**Authors:** Joanna Pakulnicka, Paweł Buczyński, Edyta Buczyńska, Edyta Stępień, Agnieszka Szlauer-Łukaszewska, Robert Stryjecki, Aleksandra Bańkowska, Vladimir Pešić, Ewa Filip, Andrzej Zawal

**Affiliations:** 1 Department of Ecology and Environmental Protection, Faculty of Biology and Biotechnology, University of Warmia and Mazury in Olsztyn, Olsztyn, Poland; 2 Department of Zoology and Nature Protection, Maria Curie-Skłodowska University, Lublin, Poland; 3 Departament of Zoology and Animal Ecology, University of Life Sciences, Lublin, Poland; 4 Institute of Marine and Environmental Science, Centre for Molecular Biology and Biotechnology, University of Szczecin, Szczecin, Poland; 5 Institute of Biology, University of Szczecin, Szczecin, Poland; 6 Department of Biology, University of Montenegro, Podgorica, Montenegro

**Keywords:** Coleoptera, Biodiversity, Landscape factors, Environmental factors, Species distribution, river, Sequentiality, RCC

## Abstract

The main goal of the study was to recognize the mechanisms underlying assemblage structuring of aquatic beetle fauna inhabiting a medium-sized, lowland river exposed to anthropogenic pressures. An attempt was made to identify the impact of numerous abiotic factors on how beetle communities are formed, with particular emphasis on geomorphological and landscape-related factors, which tend to be omitted from many studies of aquatic organisms. Our intention was to refer the results of our study to the general assumptions of the River Continuum Concept. Field studies were conducted in 2010, at 13 sites located along the Krąpiel River (north-western Poland). In total, 3,269 beetles were captured, representing 120 species and five ecological groups: crenophiles, rheophiles, rheobionts, stagnobionts a and stagnobionts b, which differ in environmental preferences. The core of the identified fauna was composed of stagnobionts, while rheophiles and rheobionts accounted for only 20% of the entire collected material. The formation of beetle assemblages was affected both by local factors, with an impact on aquatic environments, and by geomorphological factors, influencing a larger catchment. This was reflected in the high degree of conformity between dendrograms presenting similarities in the fauna at the studied sites, including the clustering of sites based on the abiotic factors that differentiated these sites. The presence of buffer zones, surfaces of patches denoted as “marshes” (marshland surface), “shrubs” (shrub surface), and “forests” (forest surface), and the distance to those patches seem to be the most important landscape factors affecting beetle communities. Of the factors influencing the aquatic environment, the following exerted the strongest effect: insolation, vegetation cover, presence of organic matter and BOD_5_, and anthropogenic pressure. The changes in assemblages of beetles determined in our study in the particular sections of the river course were a consequence of the effects of both internal factors and external ones, originating from the entire river’s catchment, which is in accord with the basic assumptions of the RCC.

## Introduction

Rivers constitute open systems whose functioning depends on the inflow of allochtonic organic matter, as referenced in Vannote et al. (1980), the authors of the river continuum concept. Therefore, rivers maintain various abiotic and biotic relationships with their surroundings (the basin), together forming a coherent ecological system. Floodplain river valleys encompass a great diversity of habitats, hydrological regimes and different degrees of connectiveness between aquatic habitats and the main river (Vinnersten et al., 2009; Pakulnicka & Nowakowski, 2012; Doretto, Piano & Larson, 2020; Turić et al., 2021). Numerous ecologists point out that the said dynamic equilibrium between the river and its surroundings can be distorted by various human activities, including agriculture, forestry, urban development, industry, construction of road infrastructure, etc. (Harding et al., 1998; Norris & Thoms, 1999; Doretto, Piano & Larson, 2020; Turić et al., 2021; Knehtl, Podgornik & Urbanič, 2021). The deterioration of water quality, which is a likely consequence, frequently decreases biological diversity of aquatic life (Vinson & Hawkins, 1998).

Although there is rich literature concerning aquatic fauna, it is only in the recent decades that scientists have started to show interest in the subject of large lowland river valleys, particularly those which have retained their natural character, and in their functioning. Nowadays, ecological studies at the landscape level are undertaken increasingly often. However, ecologists typically focus on explaining relationships between the fauna inhabiting rivers and the fauna of other water bodies located within a river’s floodplain (Castella et al., 1984; Stanford et al., 1996; van den Brink et al., 1996; Stanley, Fisher & Grimm, 1997; Ward, Tockner & Schiemer, 1999; Robinson, Tockner & Ward, 2002). The major impact of the hydrological continuity of water bodies located within river floodplains on the fauna relations among numerous groups of organisms is particularly highlighted (Castella et al., 1984, 1991; Castella & Amoros, 1988; Ward et al., 2002; Reckendorfer et al., 2006; Obolewski, Gliñska-Lewczuk & Kobus, 2009; Stryjecki et al., 2016; Zawal et al., 2016e). Turić et al. (2021) draw attention to some considerable gaps in knowledge regarding river valley floodplains and their contribution to water protection, recommending that more emphasis should be laid on studies concerning the ecological structure of fauna and the processes occurring in river valleys, so as to ensure that river floodplains can be managed more efficiently to maintain the highest possible species richness. This subject is relatively rarely studied in the context of Coleoptera communities (Vinnersten et al., 2009; Pakulnicka & Nowakowski, 2012; Costea, Cojocaru & Pusch, 2013; Pakulnicka et al., 2016a, 2016b). Because of their typically rich communities as well as ecological and functional diversity, water beetles are an important group of macroinvertebrates dwelling in aquatic habitats. In view of their high sensitivity and large dispersibility, these beetles are also an excellent bioindicator of water purity and of general biodiversity of ecosystems (Pakulnicka et al., 2015; Turić et al., 2021). Undoubtedly, knowledge of the mechanisms responsible for shaping the fauna of water beetles in river valleys would contribute to a better understanding of how these ecosystems function. This is extremely important in the face of a severe threat to river valleys posed by the progressing anthropogenic pressure (Turić et al., 2021).

The main purpose of our study was to evaluate the mechanisms responsible for the formation of the structure of beetle fauna in a valley of a medium-sized, lowland river. Our intention was to answer the following questions: (1) Does the structure of the beetle fauna change along the course of the river, from its sources to the mouth? (2) Do abiotic factors influence the ecological structure of the river fauna, and if so–to what extent do they affect it? (3) Is the characteristic structure of the beetle fauna also influenced by the geomorphological and landscape features in the river valley? (4) Is it possible to distinguish among these geomorphological and landscape traits that affect the beetle fauna the ones which have an anthropogenic origin?

Following the above research objectives, we put forth these hypotheses: H1: The ecological structure of the beetle fauna changes along the course of the river; H2: Abiotic conditions in the river shape the fauna assemblages in the distinguished sections of the river course; H3: River biocenoses are formed under the influence of the environmental conditions in the river’s catchment; and H4: The structure of the beetle fauna in the river is affected by anthropogenic factors.

## Materials and Methods

### Study area and sampling sites and environmental variables

**Figure 1:**
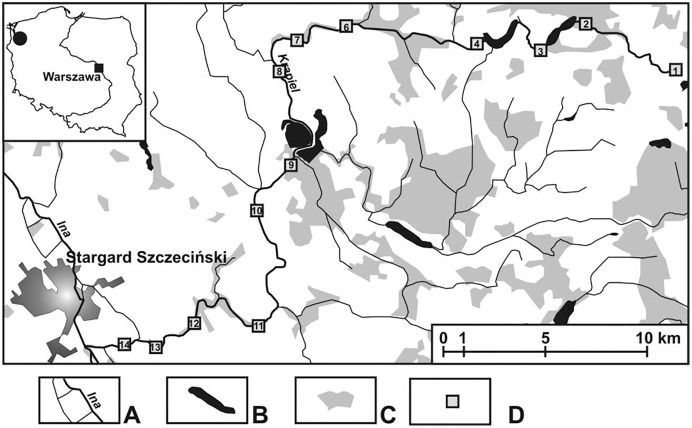
Study area showing the location of the study sites (K1–14). (A) Rivers. (B) Lakes and fish ponds. (C) Forests. (D) Study sites.

The field investigations were carried out in an area cut by a lowland, medium-sized river called the Krąpiel, situated in north-western Poland (Fig. 1). The Krąpiel River carves a deep valley in a typically agricultural area, with the prevalent share of arable land, grassland, meadows, etc., and only a negligible contribution of forests. The river valley is largely composed of areas of outstanding natural beauty. Field studies were conducted from April until October 2010. A description of the research sites is presented in Table 1. Detailed information about the study area and 13 (K1–K14) research habitats (with their mesohabitats) can be found in Zawal et al. (2015) and Pakulnicka et al. (2016b). The analyzed sites were arbitrarily assigned to three sections of the river: upper course–the initial zone, with numerous streams and their outflows (macrohabitats: K1–K6), lower course–from the river’s gorge to the mouth (macrohabitats: K12–K14), middle course–the zone between the two gorges of the river, with a wide floodplain terrace (macrohabitats: K8–K11). The following were considered: the character of the river channel, width, depth and the velocity of the river current. At all the sites, samples were collected from the river current and from pocket water spots.

**Table 1:** Characteristics of sites (macrohabitats) and sub-sites (mesohabitats) along the River Krąpiel.

Site	Sub-site	Characteristics
K1	K1/1	Current, sand, plants
K1	K1/2	Current, sand
K1	K1/3	Marginal pool, sand, mud, plants
K1	K1/4	Current, stones
K1	K1/5	Marginal pool, sand, mud
K2	K2/1	Current, stones, gravel
K2	K2/2	Marginal pool, sand
K2	K2/3	Current, sand
K3	K3/1	Marginal pool, mud, plants
K3	K3/2	Current, sand, plants
K3	K3/3	Current, sand
K3	K3/4	Current, sand, gravel
K4	K4/1	Current, mud, plants
K4	K4/2	Marginal pool, mud, plants
K5	K5/1	Current, mud, plants
K5	K5/2	Marginal pool, mud, plants
K5	K5/3	Current, peat
K5	K5/4	Current, mud, *Berula* sp.
K6	K6/1	Current, gravel
K6	K6/2	Marginal pool, sand
K6	K6/3	Current, sand
K6	K6/4	Current, plants
K6	K6/5	Current, *Fontinalis* sp.
K7	K7/1	Current, plants
K7	K7/2	Marginal pool, plants
K7	K7/3	Marginal pool, sand, mud, plants
K7	K7/4	Current, sand
K8	K8/1	Marginal pool, *Potamogeton* sp.
K8	K8/2	Marginal pool, mud
K8	K8/3	Marginal pool, *Berula* sp.
K9	K9/1	Marginal pool, plants
K9	K9/2	Current, plants
K10	K10/1	Current, Stones
K10	K10/2	Current, sand
K10	K10/3	Marginal pools, sand
K10	K10/4	Current, plants
K11	K11/1	Current, stones
K11	K11/2	Current, sand
K11	K11/3	Marginal pool, sand, mud
K12	K12/1	Marginal pool, sand, mud
K12	K12/2	Current, sand
K14	K14/1	Current, sand
K14	K14/2	Marginal pool, sand
K14	K14/3	Current, plants
K14	K14/4	Current, stones, gravel

In total, 810 samples were collected from 43 sites situated solely in lotic waters. A more detailed description of sampling, methods of determining environmental variables, selection of physicochemical parameters of water and the equipment used can be found in Zawal et al. (2015) and Pakulnicka et al. (2016b). The values of physico-chemical parameters are provided in Table 2.

**Table 2:** Values for hydrochemical parameters of water (minimum, maximum, mean and standard deviation).

Parameter	Min	Max	Mean	SD
Water temperature °C	6.4	25.1	15.2	3.20
pH	5.55	9.80	7.33	0.76
Conductivity μS cm^−1^	65	305	211	51.94
Dissolved oxygen O_2_ mg L^−1^	1.1	14.9	7.3	2.52
BOD_5_ mg L^−1^	0.0	8.5	4.73	1.38
NH_4_ mg L^−1^	0.1	3.0	0.67	0.55
NO_3_ mg L^−1^	0.2	8.8	2.24	2.08
PO_4_ mg L^−1^	0.1	1.0	0.28	0.20
Fe mg L^−1^	0.0	1.7	0.09	0.20
Turbidity mg L^−1^	0.0	304	27.99	46.78
Hardness mg L^−1^	25	288	167.88	57.15

### Ecological and statistical analyses

Species diversity was calculated using the Shannon-Wiener index (H′), the Simpson Diversity index (D) and the Pielou index (J′). To ensure adequate analysis of the beetle fauna, the identified species were divided into five groups: kr–crenophiles, re–rheophilous species, rb–rheobionts, sb–stagnobionts b, sa–stagnobionts a, (Appendix 1), according to the division proposed by Pakulnicka & Nowakowski (2012) and Pakulnicka et al. (2016b). Crenophiles are most typical of springs, rheobionts are associated with a strong river current and rheophiles have a preference for overgrown zones of rivers. In turn, type a stagnobionts comprise species found mostly in small and clean water bodies, and type b stagnobionts are typical of small, strongly eutrophic water bodies.

The Kruskal-Wallis one-way ANOVA (H) test was used to determine the significance of differences in the number of individuals, number of species and H′, and J′ in individual sampling sites (macrohabitats), as well as in the densities and percentages of the ecological components distinguished in individual habitats and sampling sites. The Spearman correlation coefficient (R_*s*_) served to determine the relationships between (1) the type of a section of the river course and the ecological components, (2) the type of habitats in the river and the ecological components, (3) the type of habitats in the river and section of the river course, and (4) individual ecological components. Correlation strength was determined after Sokal & Rohlf (1995).

Similarities between the selected subcatchments and the actual analyses of faunistic similarities between the studied sites were determined using Biodiversity Pro v.2 software (McAleece, Lambshead & Paterson, 1997). Detailed information about this method can be found in Zawal et al. (2015) and Pakulnicka et al. (2016b). The membership of objects (sites) in clusters distinguished according to the quantitative structure of the beetle assemblages as well as the clustering of object features distinguished according to the structure were verified using the nonparametric sign test (Sokal & Rohlf, 1995). All calculations were performed in Statistica 13.1 software.

Non-metric multivariate scaling (NMDS) based on the Bray-Curtis index (Bray & Curtis, 1957) was used to assess the faunal similarities between mesohabitats representing different environments (the river’s current and pocket water spots) in the upper, middle, and lower reaches of the Krąpiel River. In addition, the hierarchical agglomeration grouping of unweighted pairs with an arithmetic mean (UPGMA) of grouping species was used to illustrate the similarity relationships between all the sites. The similarity percentage method (SIMPER) identified the species responsible for assemblage discrimination between sites located in the river’s current and in the pocket water spots, as well as among the upper, middle and lower reaches of the river. These analyses were performed using Past 3.18 software (Hammer, Harper & Ryan, 2001). Multidimensional Correspondence Analysis (MCA) was used to determine the relationships between the distinguished ecological groups of beetle communities, a section of the river course (upper, middle, lower), and different mesohabitats (current and pocket river) as sampling sites.

Relationships between the presence of specific beetle species and selected environmental parameters of the Krąpiel River valley’s water bodies were determined using the Canonical Correspondence Analysis (CCA) (ter Braak, 1986; ter Braak & Verdonschot, 1995) direct ordination method (having first performed the DCA). Only features that demonstrated no collinearity were accounted for in the analyses. The adopted statistical significance level was *p* > 0.05. Species occurring rarely, *i.e*. with fewer than 10 individuals captured, were discarded from all analyses. All calculations were performed in Canoco 4.5 software.

## Results

### General characteristics of the collected material

In total, 3,269 aquatic beetle individuals (2,939 imagines and 330 larvae) representing 120 species were found in the Krąpiel River (Table 3, Appendix 1). Species diversity at respective sites was between three and 45 species. The highest number of species (112) was found in the middle course of the river (sites K8–K11). No statistically significant differences were observed between the numbers of species present in different sampling sites (macrohabitats) representing various sections of the river course: Kruskal-Wallis test H(2, *N* = 32) = 5.024, *p* = 0.81. Differences between the sampling sites for the species diversity indices were also statistically insignificant: for *N* (abundance) – H(2, *N* = 32) = 15.35, *p* = 0.068; for H′ (the Shannon-Wiener Index) − H(2, *N* = 32) = 2.947, *p* = 0.29; and for J′ (Pielou Index) − H(2, *N* = 32) = 7.88, *p* = 0.67.

**Table 3:** The main parameters of water beetle assemblages.

Parameters	Habitats		Course of the river		
	River current	River pocket	Upper	Middle	Lower
*N*	1990 (1–398)	1369 (5–360)	2169 (1–398)	746 (95–152)	354 (4–26)
S	96 (1–44)	112 (3–45)	86 (1–45)	106 (2–36)	21 (3–9)
H′	2.507 (0.178–2.92)	0.332 (0.32–2.90)	1.555 (0.12–2.92)	1.80 (0.17–2.68)	0.897 (0.47–1.38)
J′	1.685 (0.08–0.755)	0.428 (0.09–0.82)	0.513 (0.13–0.65)	0.49 (0.04–0.64)	0.442 (0.08–0.73)

**Note:**

*N*, number of individuals; S, species richness; H′, the Shannon-Wiener Index. J′, Pielou’s evenness (the numbers in brackets represent the minimum and the maximum values).

In general, 94 species were found in the river’s flowing water, whereas 110 species were found in stagnant parts of the river. The most numerous species were: *Hydraena palustris* (33.4% of total collected material), *Agabus bipustulatus* (8.5%), *Ilybius fuliginosus* (5.2%), followed by *Laccobius minutus*, *Anacaena limbata*, *Elodes* sp., *Gyrinus substriatus*, *Haliplus fluviatilis* and *Hydrobius fuscipes*. Within the entire community, most species (86) were very scarce, with four specimens captured at the most.

The core of the fauna was composed of species related to various small water bodies. Stagnobionts a (35 species) and stagnobionts b (79 species) constituted a total of 82.1% of all captured beetles, whereby stagnobionts had the highest contribution in the quantitative structure (47% of total collected material). The ecological component most typically found in rivers accounted for only a small part of the identified fauna. Rheophiles represented by 11 species made up only 14.1% of the total number of beetles, with the most numerous species being *Ilybius fuliginosus* and *Haliplus fluviatilis*. Rheobionts included 6 species, of which the most numerous were *Hydraena riparia*, *Elmis aenea* and *Oulimnius tuberculatus*. The total contribution of the entire assemblage of rheobionts was 3.3%. The river fauna also included two crenophiles, *Agabus didymus* and *Agabus biguttatus*, with a total contribution in the number of individuals at 0.2% (Appendix 1).

**Figure 2:**
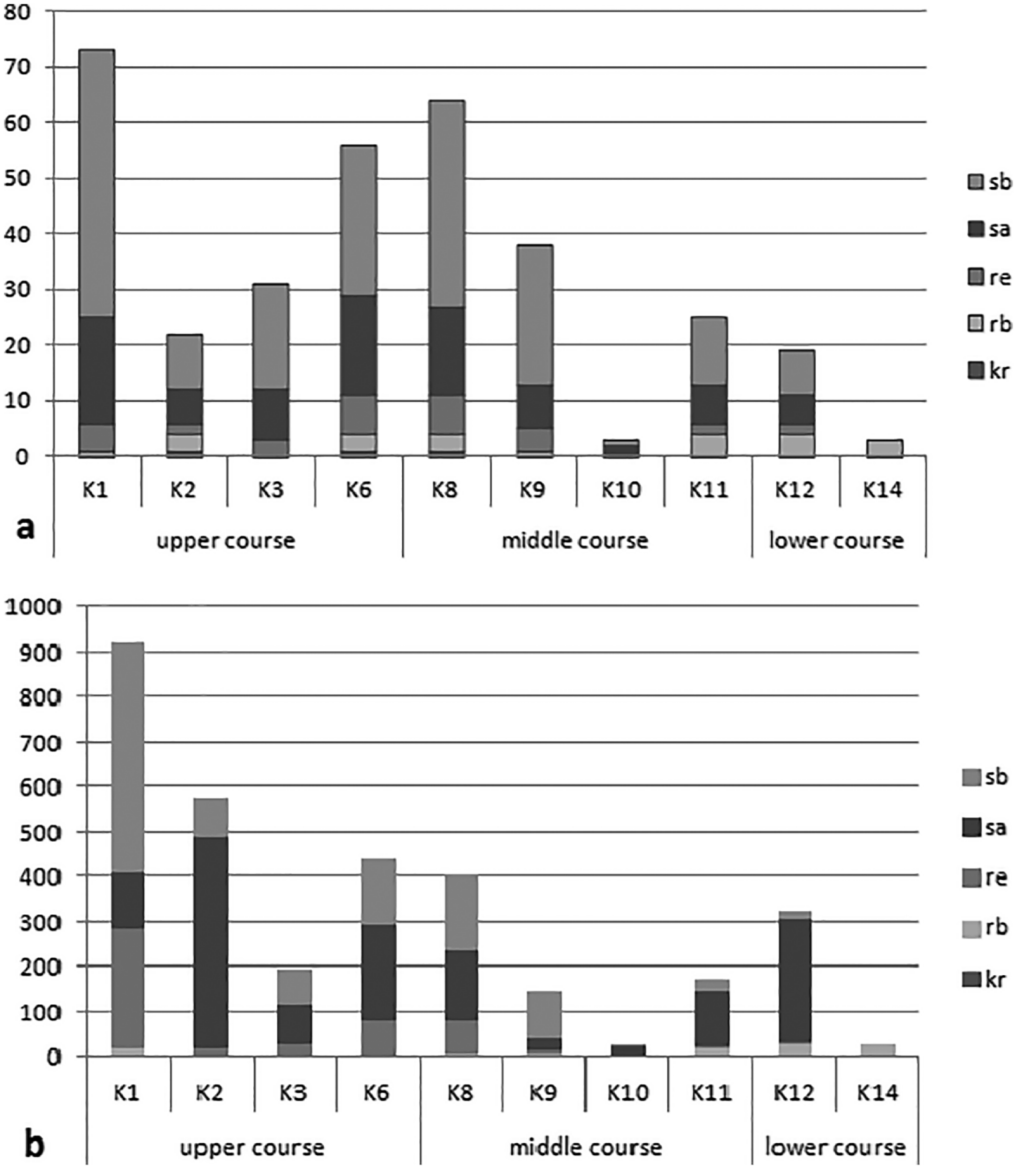
Ecological structure of the beetle fauna in each study sites in upper, middle and lower course of Krąpiel river. (A, species; B, individuals), ecological groups (sb, stagnobionts b; sa, stagnobionts a; re, rheophilous species; rb, rheobionts; kr, crenophiles) (see Appendix 1).

The analysis of the synecological structure of the beetle fauna in the habitats distinguished along the river course showed that the dominant components were stagnobionts a and b (Fig. 2). They were the most numerous groups with the greatest species richness. Only two of the ecological components showed statistically significant differences in densities between individual sampling sites in the three sections of the river course. The results from the Kruskal-Wallis test, *i.e.*, H(2, *N* = 85) = 19.64, *p* = 0.0001 for rheophilous beetles revealed significant differences between the upper and middle course (post-hoc Tukey test results: *p* = 0.008), and between the upper and lower course (post-hoc test results-*p* = 0.005), while the results from the Kruskal-Wallis test for stagnobionts b - H(2, *N* = 85) = 9.73, *p* = 0.008 showed significant differences between the upper and lower course (post-hoc Tukey test: *p* = 0.008). The remaining components showed no significant differences in densities between the three sections of the river course either for stagnobionts a H(2, *N* = 85) = 1.47, *p* = 0.47 or for crenophiles H(2, *N* = 85) = 1.01, *p* = 0.60 (Fig. 2).

A statistically significant correlation was noted between sections of the river course and the share of rheophiles (R_S_ = −0.48, *p* = 0.00003) and of stagnobionts b (R_S_ = −0.322, *p* = 0.003) (Table 4). However, stagnobionts b were found to be most abundant in the upper and middle sections of the river, and were the least numerous in the lower reaches. In turn, stagnobionts b and rheophiles made up the largest quantitative share in the fauna of beetles collected in the upper and middle sections of the river (Table 4, Fig. 2), as indicated by the above results of the Spearman correlation. At the same time, both of these ecological groups showed high mutual correlation (R*s* = 0.557) (Table 4). Both prefer water pocket habitats with ponding water, while stagnobionts b and rheobionts avoid such places (negative correlation) and prefer habitats in the river current.

**Table 4:** Results of correlations analysis (R Spearman) between the distinguished mesohabitats and ecological groups of beetles.

Mesohabitats and ecological groups	Valid *N*	Spearman R	T( *N* − 2)	*p-*value
**Course of river (upper – middle – lower)**				
crenobionts	85	0.135	1.244	0.216
rheobionts	85	0.203	1.889	0.062
rheophiles	**85**	**−0.480**	**−4.988**	**0.000003**
stagnobionts a	85	0.101	0.926	0.357
stagnobionts b	**84**	**−0.322**	**−3.078**	**0.002**
**Current river**				
crenobionts	85	0.068	0.6267	0.532
rheobionts	**85**	**0.260**	**2.4534**	**0.01615**
rheophiles	85	−0.144	−1.3307	0.186
stagnobionts a	**85**	**0.282**	**2.6778**	**0.008**
stagnobionts b	**84**	**−0.263**	**−2.4739**	**0.015**
**River pocket**				
crenobionts	85	−0.031	−0.2818	0.778
rheobionts	85	−0.130	−1.1989	0.233
rheophiles	**85**	**0.416**	**4.1744**	**0.00007**
stagnobionts a	85	0.119	1.0989	0.2749
stagnobionts b	**84**	**0.551**	**5.9860**	**0.000001**
**Ecological groups**				
crenobionts & rheobionts	85	−0.144	−1.3304	0.187
crenobionts & rheophiles	85	−0.143	−1.3175	0.191
crenobionts & a stagnobionts	85	0.159	1.4668	0.146
crenobionts & b stagnobionts	84	−0.066	−0.6062	0.546
rheobionts & rheophiles	85	0.075	0.6911	0.491
rheobionts & a stagnobionts	85	0.044	0.4078	0.684
rheobionts & b stagnobionts	84	−0.1470	−1.3476	0.181
rheophiles & a stagnobionts	85	0.168	1.5615	0.122
rheophiles & b stagnobionts	**84**	**0.557**	**6.0803**	**0.000002**
a stagnobionts & b stagnobionts	84	0.164	1.5109	0.134
**Habitats**				
current river & course	85	0.009	0.0862	0.931
current river & river pocket	**85**	**−0.842**	**−14.2413**	**0.000002**
river pocket & course	85	0.134	1.2384	0.219
river pocket & current river	**85**	**−0.842**	**−14.2413**	**0.000001**

**Note:**

Valid *N* means number of important cases. Significant values (*p* < 0.05) are reported in bold.

### Habitat clustering based on identified features and faunistic similarity

**Figure 3:**
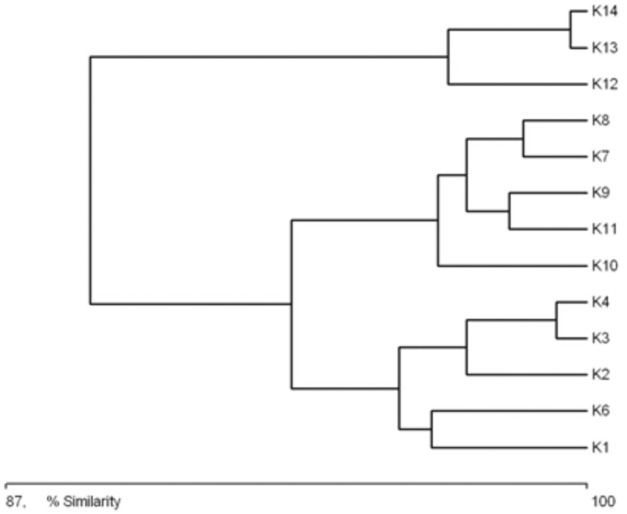
Graph showing the similarities between sampling sites (K1–14) due to the catchment’s parameters (landscape scale) (see also at Table 8).

A clear tendency emerges for the catchments of successive sites along the length of the river to be grouped according to their characteristics (Fig. 3). The first cluster consists of the catchments of sites situated in the upper course of the river (sites K1–K6). The greatest similarity within this aggregation is between the catchments of sites K4 and K3. The next group consists of the catchment areas of the sites located in the middle course of the river (K8–K11). The greatest similarity within this cluster was noted between the catchments of sites K7 and K8. The third aggregation is composed of the catchment areas of the sites in the lower course of the river (K12–K14). Within this cluster, the greatest similarity was noted between the catchments of sites K13 and K14. The main catchment parameters grouping the sites were distance from the springs, surface area of the catchment, and the river gradient in this catchment area.

**Figure 4:**
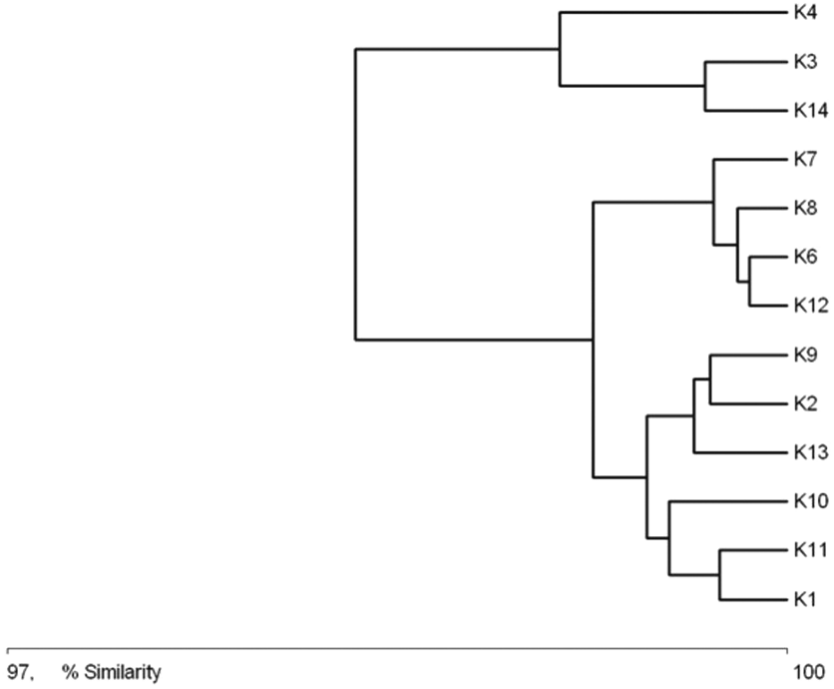
Graph showing the similarities between sampling sites (K1–14) due to the physical and chemical parameters (macrohabitat scale) (see also at Table 8).

Figure 4 shows similarities between the physical and chemical parameters of water at different sampling sites (macrohabitats). Three clusters are visible in the diagram: cluster 1, consisting of sites K4, K3 and K14; cluster 2, with sites K7, K8, K6, and K12; and cluster 3, with sites K2, K9, K13, K10, K11 and K1. Cluster 1 is characterized by an elevated nutrient content, low oxygen concentration and the highest temperature of all the sites. Cluster 3 comprises sites where the water was the cleanest, with high oxygen concentration, low NH_4_, NO_3_, PO_4_ and BOD_5_. Cluster 2 comprises sites where the water’s chemical parameters achieved intermediate values.

**Figure 5:**
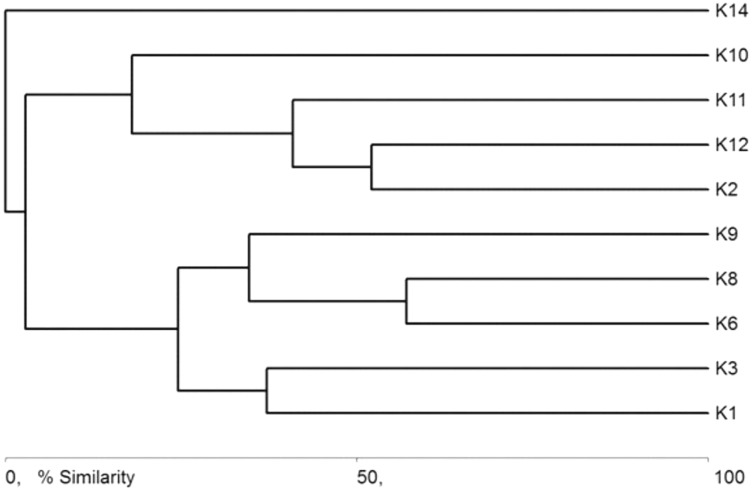
Graph showing faunistic similarities of water beetles between the study sites (K1–14).

The general specification of similarities between the fauna in the analyzed sites indicates that the maximum similarity value among those used for clustering was moderate and equaled 57% (Fig. 5). The identified habitats clearly fell into three clusters. Cluster 1 included sites located in the upper course of the river (K1–K9), among which the highest similarity appeared between sites K6 and K8, with 28 species in common. The most numerous were *Hydraena palustris*, *Ilybius fuliginosus*, *Haliplus fluviatilis*, *Hyphydrus ovatus* and *Acilius canaliculatus*. In the entire cluster, 10 species common for all habitats were found. Cluster 2 included site K2 and sites K10–K12, located in the lower course of the river. The highest similarity in this cluster (52.1%) was found between sites K2 and K12, which shared six species, including the abundant *Hydraena palustris*. The process of assigning sites to clusters based on results of the clustering according to similarities in the quantitative structure of beetle assemblages (Fig. 5) is consistent with the process of classifying sites in groups (clusters) according to their specific landscape properties (Fig. 3) (sign test: Z = 0.5, df = 13, *p* = 0.62), and with the assignment of sites to groups (clusters) differentiated according to hydrochemical parameters (Fig. 4) (sign test: Z = 0.45, df = 13, *p* = 0.68).

**Figure 6:**
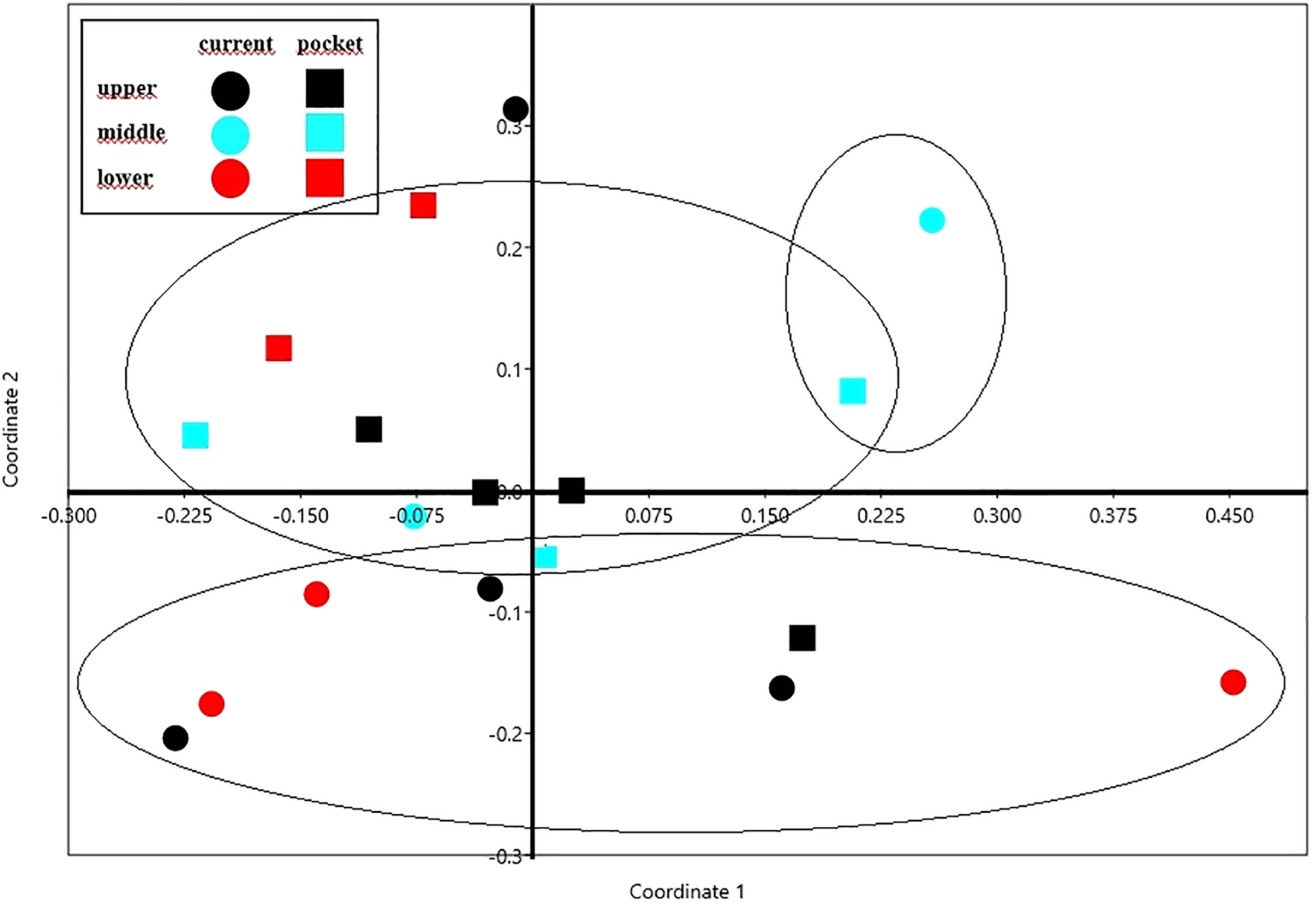
The non-metric multivariate scaling (NMDS) plot showing the beetle fauna similarities at the sites (K1–14) located in the upper, middle and lower reaches of the river in two types of habitats–in the current and in pocket water spots. Stress value: 0.146. Calculations based on the Bray-Curtis formula.

**Figure 7:**
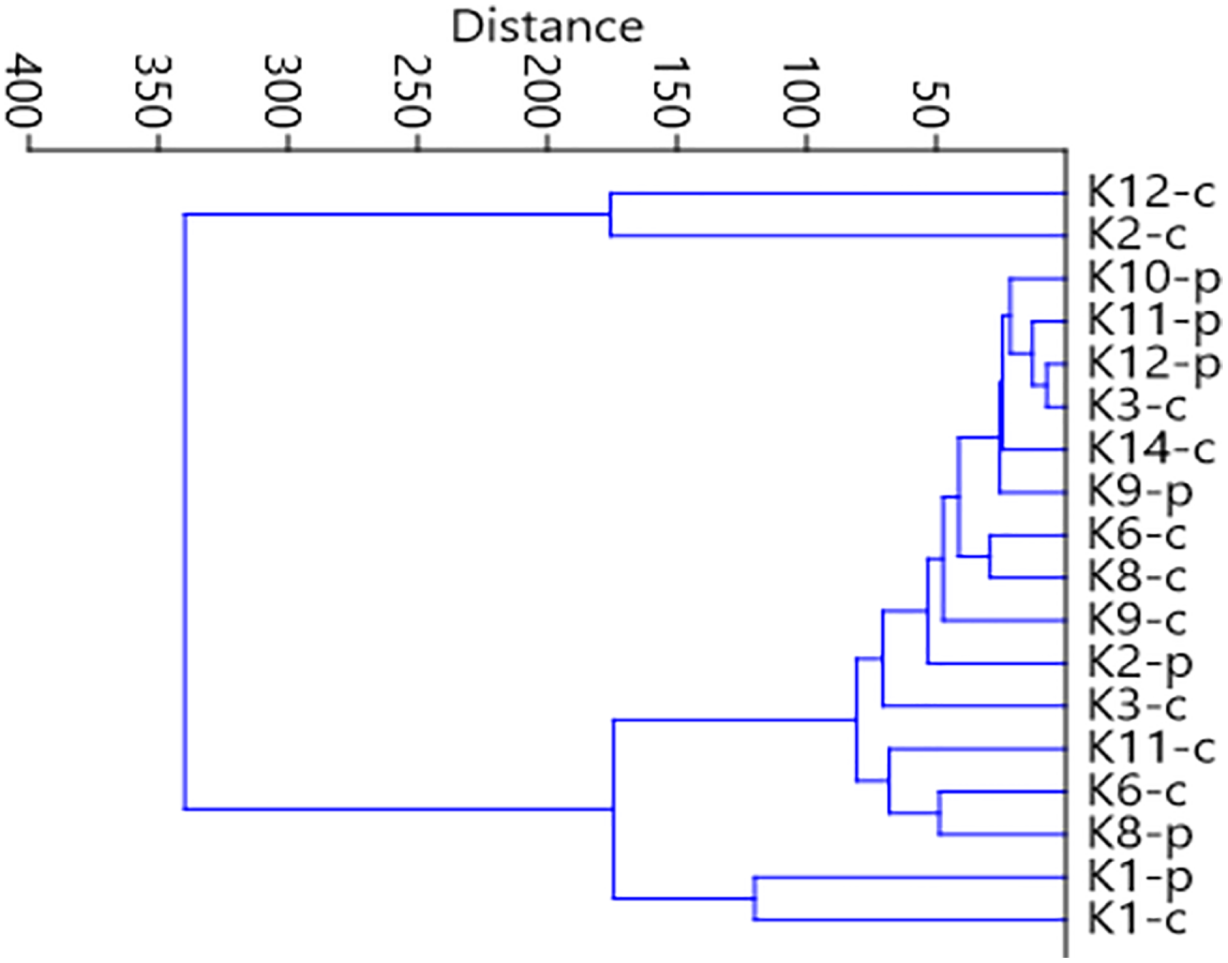
Graph showing faunistic similarities of water beetles between the distinguished mesohabitats.

The NMDS diagram shows the beetle fauna similarities at the sites located in the upper, middle and lower reaches of the river in two types of habitats-in the river’s current and in pocket water spots (Fig. 6). As a rule, the first axis (Eigenvalue: 0.341) separates the sites located in the lower course of the river, with the exception of site K14 located in the current (first and fourth quarter of the diagram). The second axis (Eigenvalue: 0.228), in turn, separates the places located in the current from the others. Faunistic similarities between the distinguished mesohabitats can also be seen in the following diagram (Fig. 7).

The SIMPER analysis (Table 5) showed high dissimilarity between beetle communities in mesohabitats located in the river’s current and pocket water. *Hydraena palustris* (30.8%) and *Agabus bipustulatus* (5.75%) made the greatest contribution to this difference. These two species also contributed the most to dissimilarities between the sites localized in particular sections of the river course (28.2% and 5.96%, respectively). Other species, especially *Elodes* sp. *Gyrinus substriatus* as well as *Ilybius fuliginosus* and *Colymbetes fuscus*, were also of great importance for the differences between the discussed habitats.

**Table 5:** Results of a similarity percentage (SIMPER) analysis between the beetle assemblages at the sampling sites located in two habitats (current and stagnant) in the upper, middle and lower reaches of the river.

Sites (habitat types)	Oad %	Species responsible the most for dissimilarity						
		Species	Av. dissim.	Contrib. %	Cumulative	Mean 1	Mean 2	Mean 3
Current & river pocket	86.47	*Hydraena palustris*	26.63	30.79	30.79	104.0	16.9	–
		*Agabus bipustulatus*	4.92	5.69	36.48	15.9	15	–
		*Elodes* sp.	3.10	3.59	48.64	16.0	4.0	–
		*Gyrinus substriatus*	2.57	2.97	51.61	1.56	5.56	–
Upper & middle & lower	86.43	*Hydraena palustris*	24.4	28.23	28.23	74.5	23.0	76.0
		*Agabus bipustulatus*	5.15	5.96	34.19	34.3	0.8	0.0
		*Ilybius fuliginosus*	4.45	5.15	39.33	16.9	6.4	0.4
		*Colymbetes fuscus*	3.77	4.36	43.69	0.5	11.8	0.0

**Note:**

Oad%–the overall average % of dissimilarity.

### Dependencies between environmental factors and beetle groups along the river course

**Figure 8:**
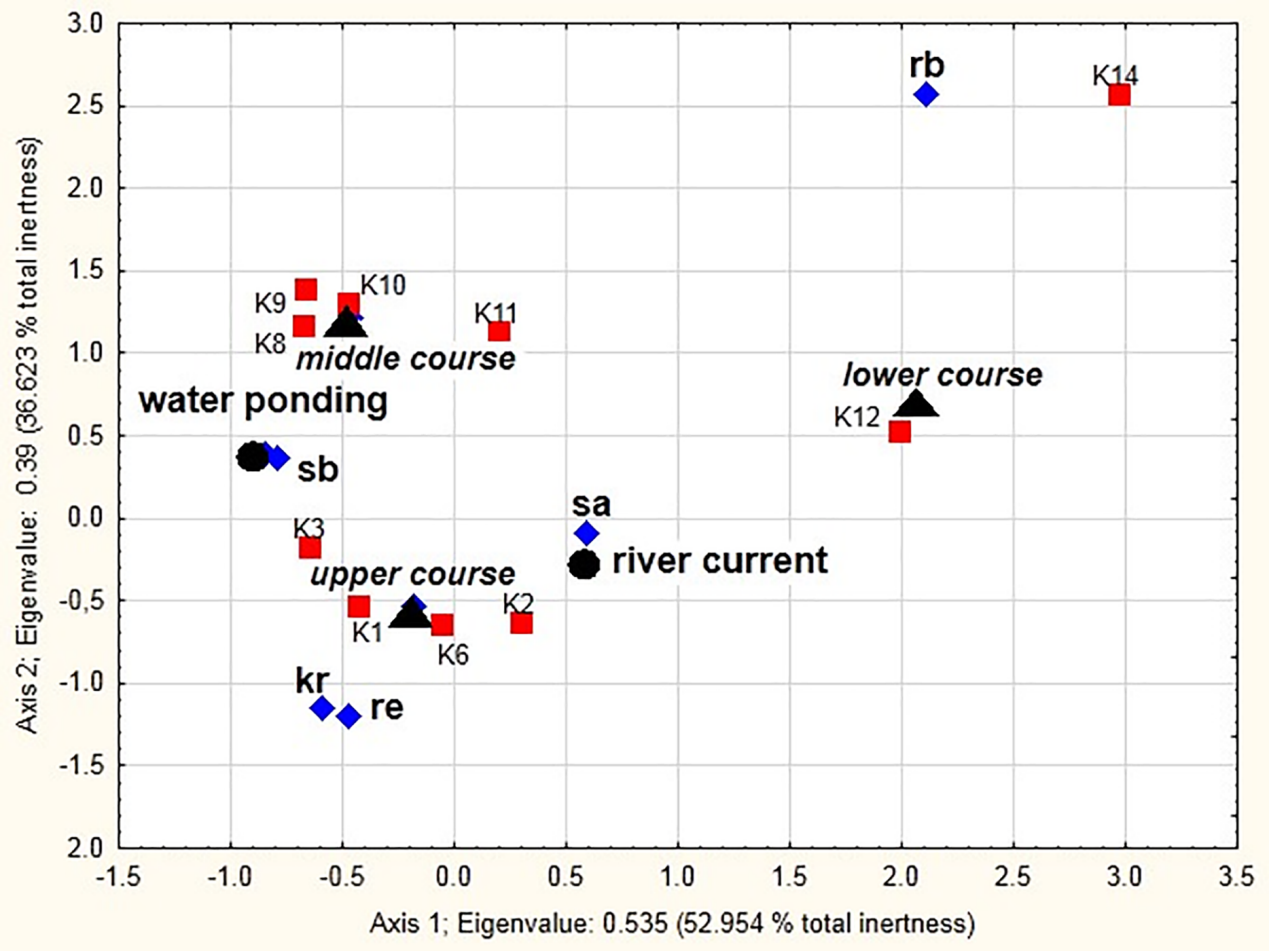
Multidimensional Correspondence Analysis (MCA). The relationships between distinguished ecological groups (sb, stagnobionts b; sa, stagnobionts a; re, rheophilous species; rb, rheobionts; kr, crenophiles; marked with a diamond) of beetle communities, a section of the river course (upper, middle, lower, marked by a triangle), and different mesohabitats (current and pocket river, marked with a circle) as sampling sites (marked with a square) along the first and second MCA axis.

Significant correlation (*x*^2^ = 24116.7, df = 81, *p* < 0.001) between the distinguished ecological groups, the river course sections, and different mesohabitats (the current and pocket water spots) as sampling sites is shown in the MCA diagram (Fig. 8). The two dimensions (the vertical and horizontal axes) together explain 89.58% of the total chi-squared statistic (total inertia) (Table 6). The analysis confirmed strong positive correlations between stagnobionts a and rheobionts and mesohabitats with the river current (particularly in the lower and upper course) (see Appendix 2). Stagnobionts b showed a strong relationship with mesohabitats in the pocket water spots with water ponding, especially in the middle and upper course of the river. Crenophilous species and rheophiles showed marked affinity for habitats with water ponding in the upper course.

**Table 6:** Results of multidimensional correspondence analysis (MCA).

Number of dimensions	Singular values	Eigenvalues	Percent of interia	Cumulative percent	Chi squares
1	0.731840	0.535590	52.95387	52.9539	565.489
2	0.622800	0.387880	36.62343	89.5773	409.142
3	0.578426	0.334576	4.33899	91.9163	352.788
4	0.577350	0.333333	4.28571	98.2020	351.673
5	0.558011	0.311377	1.79844	100.0000	328.030

**Note:**

The first two dimensions account for approximately 89.58% of the total variation.

### Presence of beetles *vs* habitat and landscape factors

The DCA of the distribution of beetles showed that the length of the gradient represented by the first ordination axis was 4.257, which justifies using the direct ordination analysis CCA to determine the relationship between the occurrence of species and the researched environmental parameters of the Krąpiel River (ter Braak, 1986; ter Braak & Verdonschot, 1995). The Eigenvalues indicate that the gradient represented by the first ordination axis significantly differentiates the presence of species (0.796), as its specific value exceeds 0.5. The first axis explains 12.5% of the variability of the beetle species composition, while for the second axis this value is 7.8% (Table 7).

**Table 7:** Results of detrended correspondence analysis (DCA).

Axes	1	2	3	4	Total inertia
Eigenvalues	0.796	0.502	0.348	0.272	6.383
Lengths of gradient	4.257	5.013	4.491	4.585	
Cumulative percentage variance of species	12.5	20.3	25.8	30.0	
Sum of All eigenvalues					6.383

**Figure 9:**
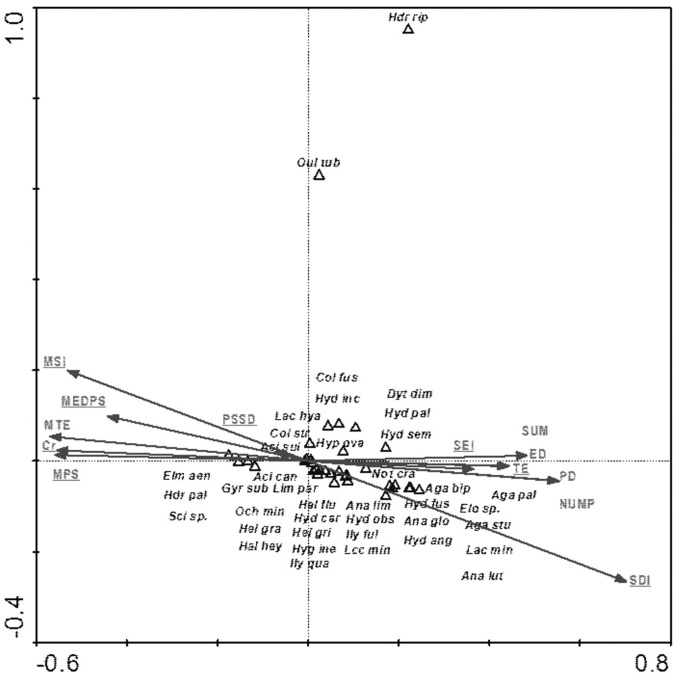
The CCA ordination plot of occurrence of water beetles in relation to the characteristics of landscape buffer zones along the first and second CCA axis. Full species names are given in Appendix 1. Full characteristics names are given in Table 8. Statistically significant variables are underlined (exact *p* values are given in Table 8).

Among the analyzed landscape characteristics of the buffer zones, the following variables were of statistical significance: SDI (Shannon patch diversity index), MEDPS (median patch size), MSI (median shape index), SEI (Shannon evenness index), PSSD (patch size standard deviation), TE (total edge length) and Cr (density) and MPS (mean patch size) (Table 8, Fig. 9). These variables explain 35.8% of the total variability of the fauna species composition. The strongest relationship with the analyzed factors (mainly SE-positive correlations; SDI and TE-negative correlations) is shown for *Hydraena riparia* and *Oulimnius tuberculatus*. The remaining species demonstrate a negative although average and weak relationship with most of the analyzed landscape factors. Results of the Monte Carlo permutation test (499 permutations) for statistically significant relationships between species and environmental factors are shown in Table 8.

**Table 8:** Results of the Monte Carlo permutation test (499 permutations) for statistically significant relationships between species and environmental factors.

Parameter	Var *N*	Lambda *A*	*p* value	*F*
**CCA–Relationship between distribution of beetles and landscape characteristics of buffer zones (Fig. 9)**				
SDI–Shannon patch diversity index	12	0.51	**0.002**	5.49
MEDPS–median patch size	3	0.27	**0.002**	2.94
MSI–median shape index	10	0.30	**0.002**	3.50
SIE–Shannon evenness index	13	0.18	**0.008**	2.15
Cr–density	11	0.35	**0.002**	4.32
PSSD–patch size standard deviation	2	0.23	**0.002**	2.92
TE–total edge length	6	0.39	**0.002**	5.28
MPS–mean patch size	1	0.06	0.500	0.81
**CCA–Relationship between distribution of beetles and patch characteristics of buffer zones (Fig. 10)**				
MCA (9)–average patch size: meadows and pastures	9	0.64	**0.002**	7.06
L/D (16)–relationship between surface area and edge length m2/m: fens	7	0.37	**0.002**	4.28
CA (18)–impact of total surface area of patches of a given class: water bodies	8	0.28	**0.004**	3.37
MCA (15)–willow thickets	6	0.28	**0.020**	3.38
PD (3)–patch density: migration areas	3	0.22	**0.002**	2.91
MSI (7)–median shape index: arable land	5	0.29	**0.002**	3.85
MSI (5)–median shape index	4	0.12	0.066	1.69
PD (2)–patch density	2	0.08	0.346	1.11
**CCA–Relationship between beetle distribution and catchment surface area and surface area of individual patches in catchment (Fig. 11)**				
a field–surface area of arable land	5	0.27	**0.002**	2.77
a cat Cu–distance from springs	1	0.27	**0.006**	2.88
C–density	11	0.28	**0.002**	3.06
a marsh–surface area of marshland	8	0.35	**0.002**	4.08
river–river area	9	0.26	**0.010**	3.03
build-up–surface area of built-up land	6	0.24	**0.002**	3.01
forests–forest area	3	0.23	**0.002**	2.87
shrubs–surface area of shrubs	10	0.23	**0.002**	3.01
a cat–catchment area	2	0.18	**0.066**	2.53
fields–surface area of arable land	5	0.27	**0.002**	2.77
**CCA–Relationship between distribution of beetles and river gradient, catchment distance from springs and distance of individual patches within the catchment from the river (Fig. 12)**				
d wast–distance from wast	9	0.54	**0.002**	5.88
d shrub–distance from shrubs	8	0.36	**0.002**	3.97
d marshes–distance from marshes	5	0.18	**0.016**	2.18
d source–distance from source	2	0.15	0.054	1.65
river gr–river gradient	1	0.30	**0.002**	3.67
d build-up–distance from built-up land	6	0.18	**0.034**	2.20
d forests–distance from forest	3	0.13	0.116	1.71
d fields–distance from arable land	4	0.27	**0.002**	3.57
d mead–distance from meadows	7	0.20	**0.048**	2.70
**CCA–Relationship between beetle distribution and river bed structure (Fig. 13)**				
insolati–insolation level	2	0.52	**0.002**	5.58
organic–substrate type	3	0.22	**0.018**	2.48
plants–degree of aquatic plant cover	7	0.17	**0.024**	1.83
velocity–water velocity (m/s)	1	0.16	0.058	1.79
M–mean sediment grain size	5	0.14	0.070	1.58
W–sediment sorting	6	0.06	0.730	0.77
**CCA–Relationship between beetle distribution and hydrochemical parameters of water (Fig. 14)**				
BOD (mg O2 dm– 3)–biological oxygen demand	11	0.41	**0.002**	4.30
cond.–electrolytic conductivity (μS cm^−1^)	4	0.30	**0.002**	3.26
Fe–concentration of iron ions (mg dm^−3^)	8	0.17	**0.026**	1.95
NO_4_–concentration of nitrogen (mg dm^−3^)	6	0.15	0.134	1.70
temp.–temperature (°C)	3	0.14	0.068	1.49
PO_4_–concentration of phosphate ions (mg dm^−3^)	7	0.10	0.226	1.23
turbidity–water turbidity level	9	0.11	0.206	1.24
O_2_–oxygen saturation kontent (%)	1	0.12	0.126	1.37
pH–pH value	2	0.11	0.172	1.29
hardness–hardness degree	10	0.08	0.578	0.87
NH_4_–concentration of ammonium (mg dm^−3^)	5	0.07	0.688	0.78

**Note:**

Var. *N*, number of random variables; CCA, canonical correspondence analysis. Significant values (*p* < 0.05) are reported in bold.

**Figure 10:**
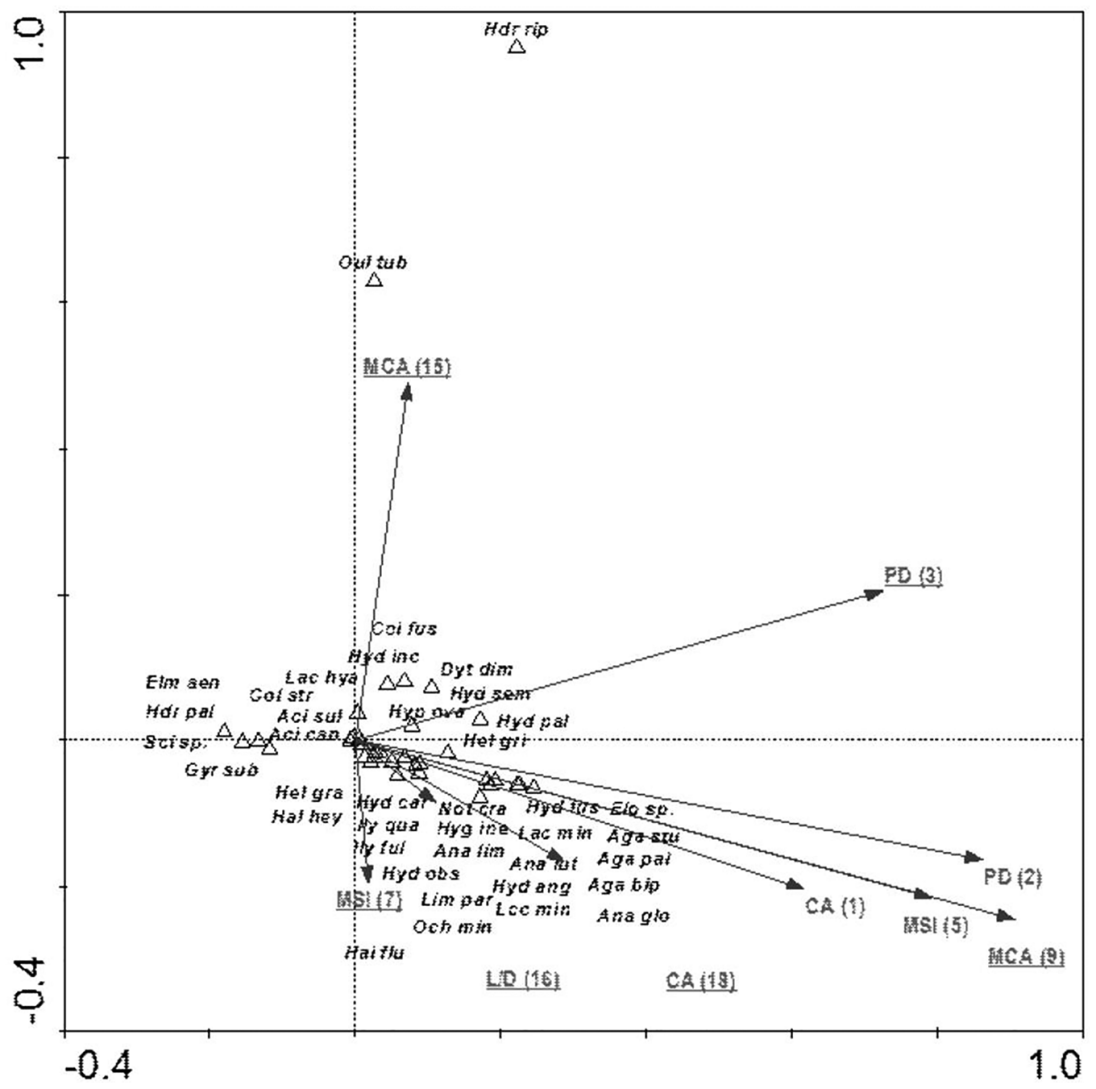
The CCA ordination plot of occurrence of water beetles in relation to the characteristics of patches in the buffer zones along the first and second CCA axis. Full species names are given in Appendix 1. Full names of characteristics are given in Table 8. Statistically significant variables are underlined (exact *p* values are given in Table 8).

The species composition of beetle fauna also depended on the characteristics of individual patches within the zones. The highest importance here is that of the impact of the total surface area of patches of a given class: water bodies – CA (18); relationship between surface area and edge length m^2^/m: fens – L/D (16); average patch size: meadows and pastures – MCA (9), willow thickets: – MCA (15); median shape index: arable land – MSI (7); patch density: migration areas – PD (3) (Fig. 10). The variables applied in the ordination explain 43.8% of the total variability of beetle fauna species composition. Most species showed positive correlation with environmental parameters. Positive correlation was found between the MCA (15) and PD (3) *vs Hydraena riparia* and *Oulimnius tuberculatus*, and a much weaker correlation was determined between these two factors and an assemblage consisting of *Colymbetes fuscus*, *Hydroporus incognitus*, *Dytiscus dimidiatus*, *Hydaticus seminiger*, *Hydroporus palustris* and *Hyphydrus ovatus*. Other factors positively affected 21 species representing various ecological components. The strongest relationships were found between patches of meadows/pastures or patches of land and a relatively non-uniform assemblage of beetles comprising mainly *Agabus paludosus*, *A. bipustulatus*, *A. sturmi* and *Elodes* sp. and *Anacaena globulus* (Table 8, Fig. 10).

**Figure 11:**
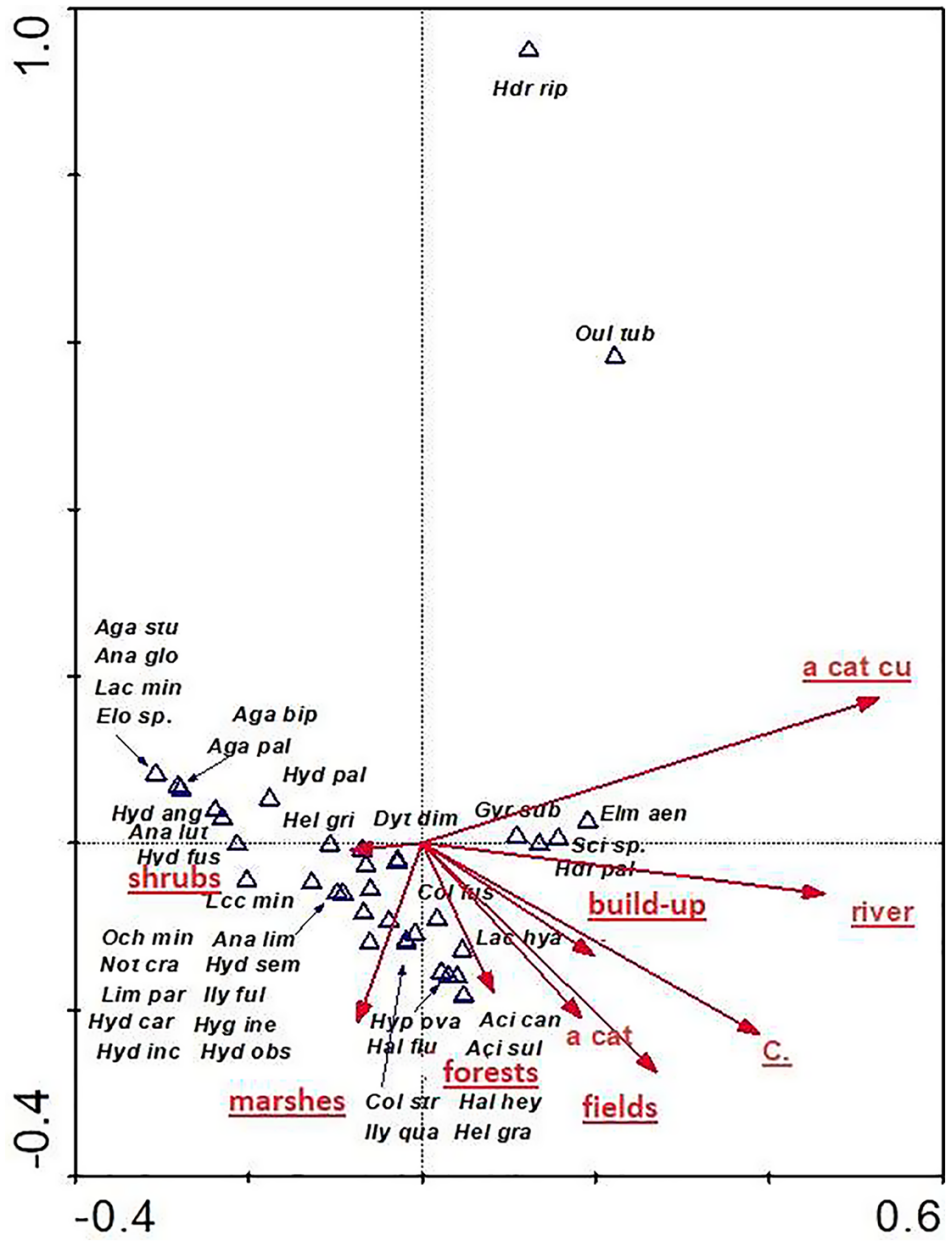
The CCA ordination plot of occurrence of water beetles in relation to the catchment surface area and surface area of individual patches within the catchment along the first and second CCA axis. Full species names are given in Appendix 1. Full names of characteristics are given in Table 8. Statistically significant variables are underlined (exact *p* values are given in Table 8).

The distribution of aquatic beetles was also affected by the catchment surface area and surface areas of individual patches within the catchment. Of significance were all parameters (except for “a cat”-catchment area), explaining 32.8% of total variability of beetle species composition (Fig. 11). Most species (21) showed a relationship with “shrubs” (surface area of shrubs) and “marshes” (surface area of marshland). A positive correlation was found between “a cut cu” (distance from springs) and: *Oulimnius tuberculatus*, *Hydraena riparia*, *H. palustris*, *Elmis aenea* and *Scirtes* sp. The assemblage of the remaining seven species was affected by “forests” (area of forests), “fields” (surface area of arable land), “build-up” (surface area of built-up land), as well as “river” (river area) and “C” (density) (Table 8, Fig. 11).

**Figure 12:**
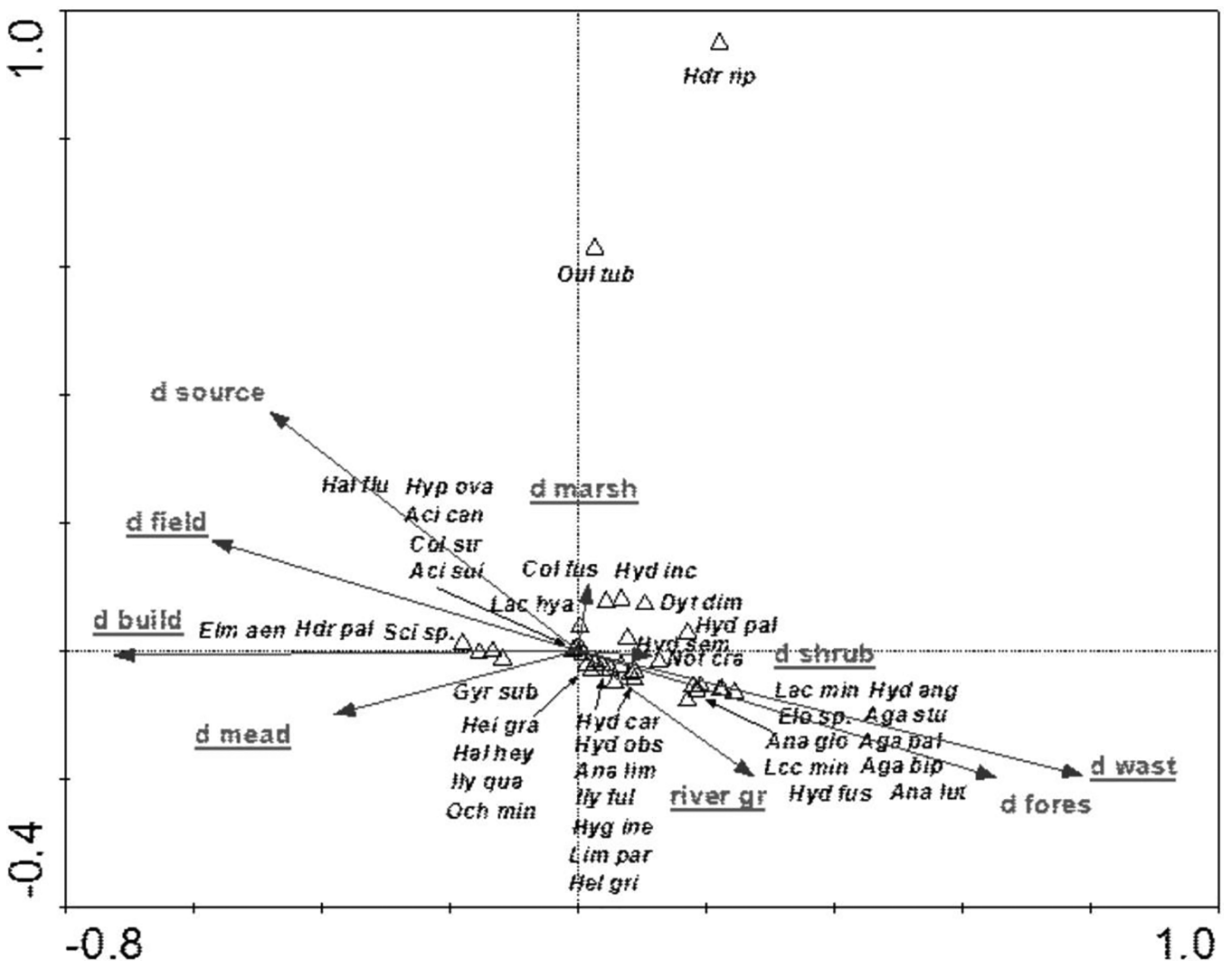
The CCA ordination plot of occurrence of water beetles in relation to the river gradient, distance of catchment from springs, and distance of individual patches in the catchment from the river along the first and second CCA axis. Full species names are given in Appendix 1. Full names of characteristics are given in Table 8. Statistically significant variables are underlined (exact *p* values are given in Table 8).

The species structure of the beetle fauna also depended on the river gradient, catchment distance from springs and distance of individual patches within the catchment from the river (Fig. 12). The variables applied in the ordination explain 36.3% of total variability. The presence of most species showed positive correlation with “d shrubs” (distance from shrubs), “d mead” (distance from meadows) and “river gr” (river gradient). In turn, “d marshes” (distance from marshes) was correlated with *Hydraena riparia*, *Oulimnius tuberculatus*, *Dytiscus dimidiatus*, *Colymbetes fuscus*, *Hydaticus seminiger*, *Hydroporus incognitus* and *H. palustris*. The only species showing positive correlation with “d fields” (distance from arable land) and with “d build-up” (distance from built-up land) were *Elmis aenea*, *Hydroporus palustris* and *Scirtes* sp. (Table 8, Fig. 12).

**Figure 13:**
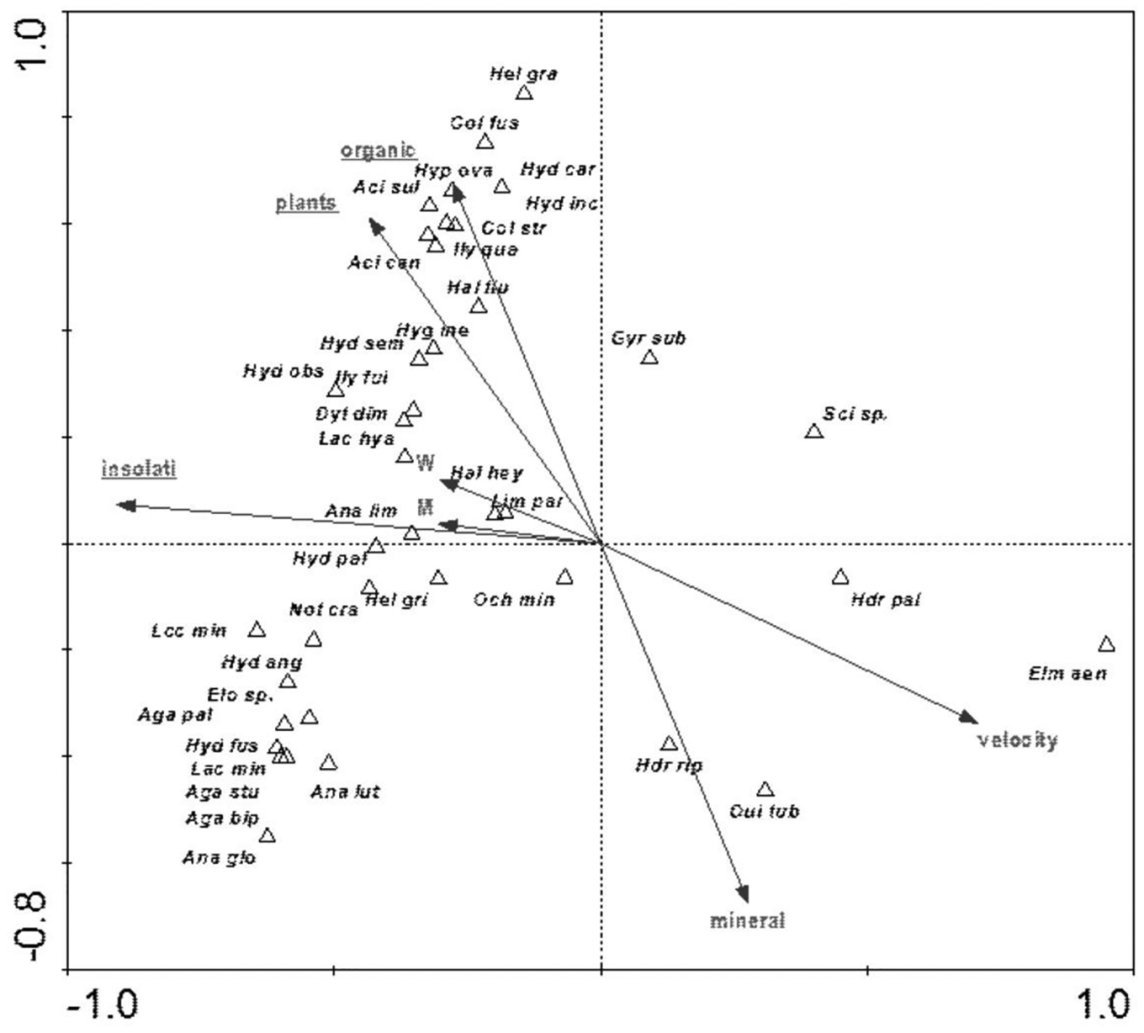
The CCA ordination plot of occurrence of water beetles in relation to the structure of the river bed along the first and second CCA axis. Full species names are given in Appendix 1. Full names of characteristics are given in Table 8. Statistically significant variables are underlined (exact *p* values are given in Table 8).

The distribution of aquatic beetles was also affected by the river bed structure: “plants”, “insolation”, “organic”, which explain 20.0% of the total species variability (Fig. 13). Positive correlation with these parameters was shown by a non-uniform assemblage composed of 19 species, whereas negative correlation emerged for *Hydraena palustris*, *H. riparia*, *Elmis aenea* and *Oulimnius tuberculatus* (Table 8, Fig. 13).

**Figure 14:**
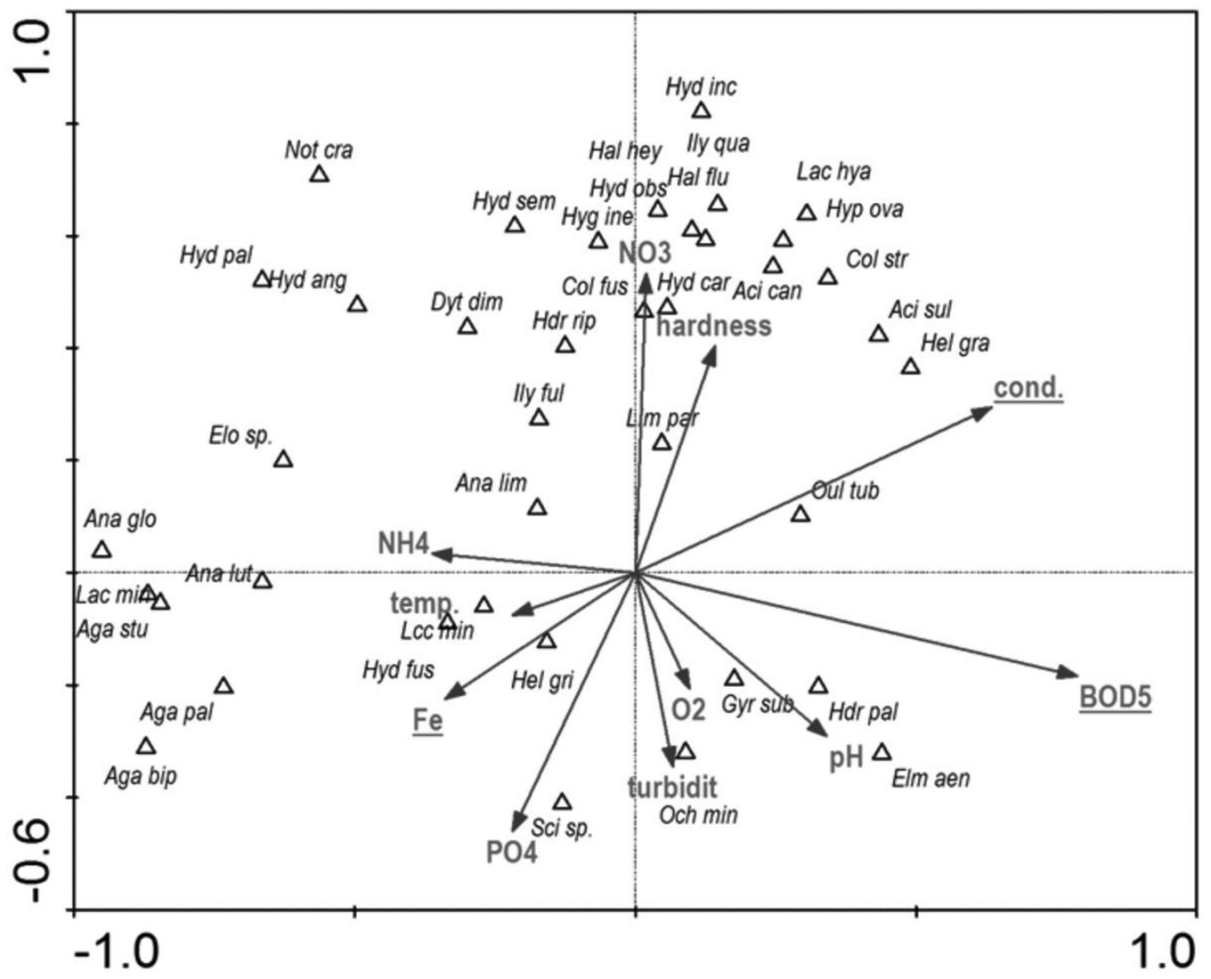
The CCA ordination plot of occurrence of water beetles in relation to hydrochemical parameters of water along the first and second CCA axis. Full species names are given in Appendix 1. Full characteristics names are given in Table 8. Statistically significant variables are underlined (exact *p* values are given in Table 8).

Among the studied hydrochemical parameters of water, the following affected the presence of beetles: BOD_5_, conductivity and Fe, explaining 27.5% of total variability of species composition (Fig. 14). Increase in BOD_5_ positively affected the presence of *Gyrinus substriatus*, *Hydraena palustris*, *Ochthebius minimus* and *Elmis aenea*. In turn, electrolytic conductivity was positively related to *Haliplus fluviatilis*, *Oulimnius tuberculatus*, *Helophorus granularis*, *Acilius sulcatus*, *A. canaliculatus*, *Colymbetes stratus*, *Ilybius quadriguttatus*, *Hydroporus incognitus* (Table 8, Fig. 14).

## Discussion

### Specific characteristics of beetle assemblages relative to the environmental conditions along the river’s gradient

The literature concerning the fauna of flowing water bodies typically refers to streams and brooks and, to a lesser extent, to larger lowland rivers. In the light of the results of studies conducted thus far on beetles inhabiting rivers (Vinnersten et al., 2009; Pakulnicka & Nowakowski, 2012; Pakulnicka & Biesiadka, 2011; Costea, Cojocaru & Pusch, 2013; Turić et al., 2021), the species richness of the Krąpiel River can be classified as very high. It represents more than 30% of all aquatic beetle species discovered in Poland (Bogdanowicz et al., 2004). Thus, a question arises why this river is specifically distinguished by such high biological diversity and what factors are involved.

According to the River Continuum Concept (RCC), a gradient of environmental conditions is observed along the main channel of the river, from the springs to the mouth of a river (Rice, Greenwood & Joyce, 2001; Doretto, Piano & Larson, 2020). This concerns the structure of the channel and the consequent hydraulic conditions, such as the steepness of a river, the river’s current, character of the river bed and organic matter. Meanwhile, the physical and chemical characteristics of river water change as well. Such changes induce the restructuring of the composition of fauna communities. The specificity and ecological conditions of a given ecosystem should be determined based on its most typical fauna components. For rivers, those are rheophiles and rheobionts. In the fauna captured in the Krąpiel River, these species accounted for no more than 20% of all captured beetles. This is considerably less than in the lowland Niemen River (Pakulnicka & Nowakowski, 2012). The remaining part are species usually related to other small water body environments (stagnobionts a), *i.e.*, dystrophic water bodies, either in forests or with poor vegetation, with more mineralized soil substrate and, in considerably fewer numbers, to eurytopic species (stagnobionts b).

The high number of rheophilic species (*e.g., Ilybius fuliginosus*, *Laccophilus hyalinus*, *Haliplus fluviatilis)*, reobionts (**e.g.*, Hydraena riparia*, *Elmis aenea* and *Oulimnius tuberculatus*) and species related to small, weakly eutrophic water bodies is characteristic of the river beetle fauna resulting from the presence of a sandy river bed. In our research, as confirmed by the CCA results, these species show strong positive correlation with electrolytic conductivity and negative correlation with BOD, which indicates that they prefer clean and well-oxygenated waters. The impact of the physical and chemical parameters of water on the formation of fauna relationships is underlined by Marchese & Ezcurra de Drago (1992), Di Sabatino, Gerecke & Martin (2000), Sanderson, Eyre & Rushton (2005), Costea, Cojocaru & Pusch (2013), Pakulnicka et al. (2015), Pakulnicka, Górski & Bielecki (2015), Pakulnicka et al. (2016a, 2016b) and others. In turn, wider or narrower belts of shoreline plants, where insolation is lower (the CCA analysis), promote the presence of species associated with heavily eutrophic waters as well as tyrphophilous species (*Colymbetes striatus, Hydroporus incognitus, H. obscurus*), for which shallow river waters with accumulated organic matter (the CCA analysis) are a habitat that imitates the conditions they find in peatland waters. The strong dependence of the fauna of beetles on the extent to which a water body is covered with macrophytes has been mentioned by Sanderson, Eyre & Rushton (2005), Pakulnicka (2008), De Szalay & Resh (2008), Hassall, Hollinshead & Hull (2011), Pakulnicka & Nowakowski (2012) and Zawal et al. (2016c, 2016d). Many authors point to the fact that the water plant cover affects beetle assemblages more that the water chemical properties do (*e.g.*, Nilsson & Söderberg, 1996; Winfield Fairchild, Faulds & Matta, 2000).

**Figure 15:**
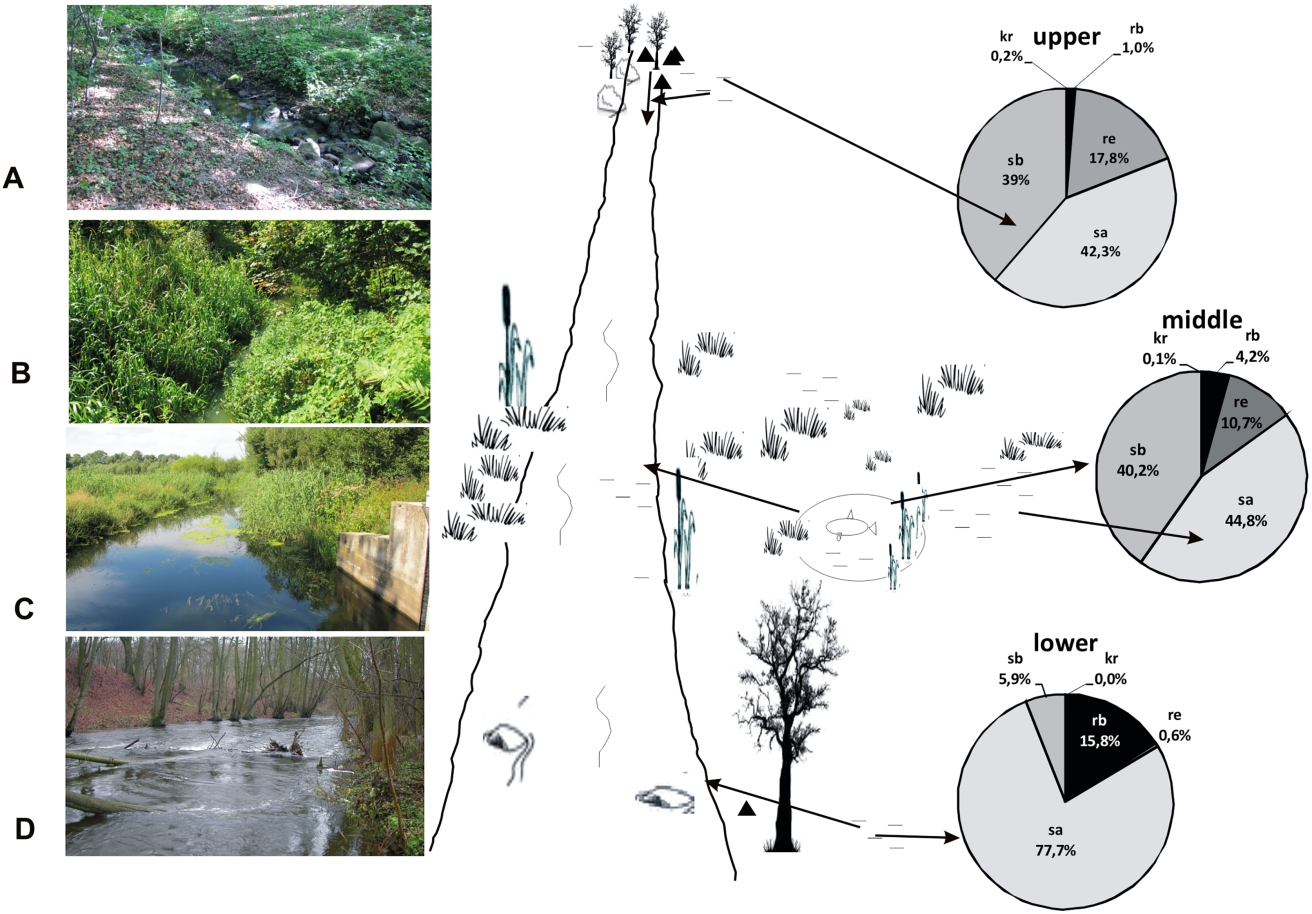
A model showing the distribution of beetles in the valley of the Krąpiel River. Ecological structure of beetles in the upper (A, B), middle (C) and lower (D) river course. Red arrows mark the directions of beetle migration, triangles mark springs, horizontal lines mark water and wetlands.

According to the RCC, we should expect that the current would become weaker in the lower course of the river, near its mouth, while more numerous pocket water spots should appear on the shallow river banks overgrown with macrophytes. However, the sequence of the habitats along the river course is disturbed in the lower course of the Krąpiel River, which is demonstrated by the NMDS analysis. In this location, the Krąpiel River flows in a narrow valley and resembles a mountain stream. Consequently, the environmental conditions present there are closer to ones in the research sites located along the upper course of the river (Fig. 15), which results in the evident dominance of rheobionts (94%), mainly *Hydraena* sp. and stagnobionts a, as displayed by the MCA analysis and the SIMPER analysis. In turn, the middle course of the river, which has characteristics of a lowland river, is dominated by stagnobionts b and rheophiles. A positive relationship between water organisms and the velocity of a river has also been demonstrated by Böttger & Martin (1995), van Der Hammen & Smit (1996), Martin (1996, 1997), Zawal et al. (2016c, 2016d). Thus, environmental factors (*e.g.*, velocity of river current, subsoil, bed depth, macrophytes, etc.) are the most important factors influencing the character of a community of beetles, which has also been suggested in studies on other groups of organisms (da Conceição, Higuti & Martens, 2017; Szlauer-Łukaszewska & Pesić, 2020). Our research allowed us to confirm the hypotheses H1 and H2, that the ecological structure of the beetle fauna changes along the course of the river, and that abiotic conditions in the river shape the fauna assemblages in the distinguished sections of the river course.

### Effect of the river basin elements on assemblages of beetles

Another assumption of the River Continuum Concept (RCC) is that a river is under a strong impact of environmental conditions prevalent in the river valley (da Conceição, Higuti & Martens, 2017; Doretto, Piano & Larson, 2020; Turić et al., 2021). For aquatic organisms, including beetles, the presence of stagnant water bodies and their character are important, which is confirmed by the dendrograms illustrating results of an analysis of habitat and fauna similarities (Figs. 3, 5). It is also supported by studies on other groups of water organisms (Sanderson, Eyre & Rushton, 2005; Stryjecki et al., 2016; Zawal et al., 2016d; da Conceição, Higuti & Martens, 2017).

We show this situation in our model (Fig. 15). The eurytopic species preferring small, more eutrophic water bodies migrate to the river seeking better habitat conditions. Most of them appear only in small numbers. Migration and movement of fauna from nearby water bodies to a river is possible owing to the high dispersion capacity of water beetles, the majority of which are capable of good or even very good flight. Migration is promoted by the close proximity of various water bodies (da Conceição, Higuti & Martens, 2017). In the Krąpiel River valley, these include springs, river broads, sedges, permanent water bodies as well as pools, oxbows and fish ponds. The high diversity of water bodies in the Krąpiel River valley is conducive to the high local species richness, as confirmed by the results of research conducted in other, yet similar areas (*e.g.*, Stanley, Fisher & Grimm, 1997; Wissinger, 1999; Junk, 2000; Tockner, Malard & Ward, 2000; Robinson, Tockner & Ward, 2002; Costea, Cojocaru & Pusch, 2013; Pakulnicka et al., 2016a, 2016b; Zawal et al., 2016a, 2016b, 2016c, 2016d). In accordance with the RCC assumptions, the impact of stagnant waters should be stronger in the lower section of a river (Doretto, Piano & Larson, 2020), interfering with the sequences of assemblages suggested by the biological continuum along the watercourse. However, the MCA showed a specific impact of eutrophic water bodies with *Glyceria* sp., for example plant overgrown oxbow lakes or artificial water reservoirs like fish ponds in the middle course of the river, which was manifested by the presence of dominant stagnobionts b, especially in water ponding sites.

The river valley means more than just water, as it also includes components of the terrestrial environment, which may constitute a certain obstacle for insects undertaking migration by air (Pakulnicka et al., 2016a, 2016b). The high importance of the terrestrial environment, particularly the landscape structure, type of landscape and geomorphology, in the formation of clusters of aquatic organism is highlighted, among others, by Richards & Host (1994), Richards, Johnson & Host (1996), Pither & Taylor (1998), Deletre & Morvan (2000), Robinson, Tockner & Ward (2002), Doretto, Piano & Larson (2020) and Turić et al. (2021). In turn, Weigel et al. (2003), Turić et al. (2021) and Knehtl, Podgornik & Urbanič (2021) underline the importance of the catchment surface area and its coverage by forests or marshlands. The CCA results indicate that the presence of buffer zones (“forests”) in the Krąpiel River valley, especially their size and composition, have a rather constraining effect on the distribution of beetles in the river, which is confirmed by negative correlations between the characteristics of these buffer zones and most of the beetle species. However, small thickets (“shrubs”) as well as marshes (“marshes”) and areas used for agriculture (“fields” and “meadows”) with periodic floods (“waste” wasteland and water bodies) have a positive influence on the beetle communities that dwell in them, mostly stagnobionts a, which have an affinity to acidified waters, *e.g.*,: *Hydroporus obscurus*, *H. incognitus*, *Colymbetes stratus*, *C. fuscus*, *Ilybius quadriguttatus* and argilophilous species, *e.g., Helophorus granularis*, *Ochthebius minimus* or *Laccobius minutus*. The beneficial effect of these landscape components on assemblages of water invertebrates has also been indicated by Deletre & Morvan (2000), Balzan (2012), Zawal et al. (2016d) and Turić et al. (2021). The results that we obtained allowed us to confirm the hypothesis H3 that river biocenoses are formed under the influence of the environmental conditions in the river’s catchment.

### Impact of anthropopressure on assemblages of beetles in the Krąpiel River

Many hydrobiologists, *e.g.*, Epele, Miserendino & Brand (2012), Miserendino et al. (2011), Buczyński et al. (2016), Doretto, Piano & Larson (2020), Turić et al. (2021) and Knehtl, Podgornik & Urbanič (2021), point to strong correlation between the use of land within the catchment and the quality of water in affected water bodies, which has further impact on the character of hydrobionts present in such environments. Allan (1995), who takes into consideration the RCC, maintains that analyses of ecological processes occurring in flowing waters should account for the heterogeneity of the environment, modified also by factors which arise form anthropogenic activities.

The catchment of the Krąpiel River is partly afforested and partly used as agricultural land. Much of it is subject to conservation under the EU Natura 2000 program, hence anthropogenic pressure is limited. Specific signs of anthropogenic pressure are clearly seen in the middle section of the river course, manifested by the presence of heavily eutrophic oxbow lakes and fish ponds over the vast floodplain river valley, while the regulated river shores and hydroengineering structures (dams and a watermill ‘a build’) along this part of the river interfere with the natural hydrology of the area (Doretto, Piano & Larson, 2020; Turić et al., 2021; Knehtl, Podgornik & Urbanič, 2021). The presence of heavily eutrophic water bodies is particularly helpful in the migration of stagnobionts b, but it also reinforces the phenomenon of potamalisation (impoundment) of the water course, which indicates the degradation of fauna typical of this section of the river (Kokavec et al., 2018). Communities of organisms in an aquatic environment are also affected by anthropogenic activities in the river channel, for example dredging (Szlauer-Łukaszewska & Zawal, 2014; Stępień et al., 2015; Buczyński et al., 2016; Dąbkowski et al., 2016; Płaska et al., 2016; Zawal et al., 2016e).

Another consequence of the anthropogenic pressure in the Krąpiel River valley is the irregularity in high water events, which implicates that the hydrological contact of the river with the water bodies in the valley has been lost. The distance from the river to other water habitats affects the structure of beetle fauna in the river (“d marshes”, “d waste”), as reflected by the CCA analysis.

The above explains why the Krąpiel River shows more stability than the less stable water bodies in the river valley. It tends to shelter more species characterized by a certain degree of stationary nature, having little or no ability to fly (Kehl & Dettner, 2007; Pakulnicka et al., 2016b). Among the fauna of the Krąpiel River, the mentioned type of beetles includes species from the genus *Haliplus* with poor flight capacity, accounting for 2.5% of all beetles, as well as *Hydroporus obscurus* and *H. rufifrons* and flightless *Hygrotus versicolor*, *Porhydrus lineatus* and *Ilybius fenestratus*. These beetles will not leave their habitats throughout their life cycle, finding optimal conditions for egg laying and wintering in their original ecosystem. They are rheophilic species, preferring clean water, and can be found in oxbows regularly flooded by high river water (Biesiadka & Pakulnicka, 2004). In the oxbows of the Krąpiel River, these species were unobserved (Pakulnicka et al., 2016b), which confirms the limited hydrological contact between the river and other water bodies located within its floodplain. Our research allowed us to prove the hypothesis H4 that the structure of the beetle fauna in the river is affected by anthropogenic factors.

## Conclusions

The lowland river Krąpiel together with its valley constitutes a fairly balanced ecological system. The presence of diverse water bodies in the river valley, however, interferes with the natural sequencing of communities as expected in line with the RCC, contributing to the heterogeneity of habitats. The Krąpiel River is inhabited by ecologically diverse fauna, dominated by stagnobionts, which migrate from other water habitats, mainly from small dystrophic water bodies. Most of the migration appears through aerial dispersion. The analyzed fauna of beetles is characterized by a relatively small share of rheobionts and rheophiles, *i.e.*, the ecological component most typical of flowing waters. The character of the fauna of the Krąpiel River is influenced by environmental factors with local impact (degree of insolation, plant cover, organic matter and BOD_5_) and ones connected with the landscape and geomorphology of the area, especially the proximity to and the surface area of buffer zones, such as marshes, shrubs and forests, in addition to various anthropogenic effects.

## Supplemental Information

10.7717/peerj.13232/supp-1 Supplemental Information 1 The quantitative statement of beetles. Abbr., abbreviation; E, synecological element (kr, crenophiles; rb, rheobionts; re, rheophiles; sa, type “a” stagnobiont; sb, type “b” stagnobiont), K1 – K 14 – macrohabitats (like in the Figure 1), C, river current; R, river pocket water; T, total; %, dominance.

10.7717/peerj.13232/supp-2 Supplemental Information 2 Results of Multidimensional Correspondence Analysis (MCA) showing the relationships between ecological groups of beetle communities (re, rb, sa, sb, kr), kind of a section of the river course (upper, middle, lower) and different mesohabitats (current and. Column. coordinates and contributions to Inertia. CN, Column number. Coordin, Coordinates. Dim, Dimension.

10.7717/peerj.13232/supp-3 Supplemental Information 3 Raw data.

## Additional Information and Declarations

### Data Availability

The following information was supplied regarding data availability: The raw measurements are available in the Supplemental File,
