# Supplementary material for: Sequentiality of beetle communities in the longitudinal gradient of a lowland river in the context of the river continuum concept
Source: PeerJ. 2022 Apr 5;10:e13232. doi: 10.7717/peerj.13232 (PMC8992647; doi:10.7717/peerj.13232)
Supplement: Supplemental Information 1 — Abbr., abbreviation; E, synecological element (kr, crenophiles; rb, rheobionts; re, rheophiles; sa, type “a” stagnobiont; sb, type “b” stagnobiont), K1 – K 14 – macrohabitats (like in the Figure 1), C, river current; R, river pocket water; T, total; %, dominance. [file peerj-10-13232-s001.docx]

Appendix 1. The quantitative statement of beetles. Abbr. – abbreviation, E – synecological element (kr –krenophiles, rb – rheobionts, re – rheophiles, sa – “a” type stagnobiont, sb – “b” type stagnobiont), K1 – K 14 – macrohabitats (like in the Figure 1), C – current river, R – river pocket, T – total, % - dominance.

| **Species** | **Abbr.** | **E** | **Macrohabitats** | | | | | | | | | | **C** | **R** | **T** | **%** |
| --- | --- | --- | --- | --- | --- | --- | --- | --- | --- | --- | --- | --- | --- | --- | --- | --- |
|  |  |  | **K1** | **K2** | **K3** | **K6** | **K8** | **K9** | **K10** | **K11** | **K12** | **K14** |  |  |  |  |
| *Gyrinus natator* | Gyr nat | sb | 1 |  |  |  |  |  |  |  |  |  |  | 1 | 1 | 0.03 |
| *Gyrinus* sp. | Gyr spe | sb |  |  |  |  | 1 |  |  |  |  |  |  | 1 | 1 | 0.03 |
| *Gyrinus substriatus* | Gyr sub | sb | 2 | 41 | 4 | 12 |  |  | 1 |  | 4 |  | 14 | 50 | 64 | 1.96 |
| *Gyrinus suffriani* | Gyr suf | sb | 1 |  |  |  |  |  |  |  |  |  |  | 1 | 1 | 0.03 |
| *Orectochilus villosus* | Ore vil | re |  |  |  |  |  |  |  | 1 |  |  |  | 1 | 1 | 0.03 |
| *Haliplus flavicollis* | Hal fla | re |  |  | 1 |  | 1 | 2 |  |  |  |  |  | 4 | 4 | 0.12 |
| *Haliplus fluviatilis* | Hal flu | re | 2 | 1 |  | 25 | 24 | 2 |  |  |  |  | 30 | 24 | 54 | 1.65 |
| *Haliplus fulvus* | Hal ful | sb |  |  |  | 3 |  |  |  |  |  |  | 3 |  | 3 | 0.09 |
| *Haliplus heydeni* | Hal hey | sb |  |  | 2 | 9 |  |  |  |  |  |  | 9 | 2 | 11 | 0.34 |
| *Haliplus immaculatus* | Hal imm | sb |  |  |  |  |  | 1 |  |  |  |  |  | 1 | 1 | 0.03 |
| *Haliplus lineatocollis* | Hal lin | re |  |  |  | 1 |  |  |  |  | 1 |  | 1 | 1 | 2 | 0.06 |
| *Haliplus ruficollis* | Hal ruf | sb | 1 |  |  |  | 1 | 2 |  |  | 1 |  |  | 5 | 5 | 0.15 |
| *Haliplus* sp. | Hal spe | sb |  |  |  |  | 1 |  |  |  |  |  | 1 |  | 1 | 0.03 |
| *Peltodytes caesus* | Pel cae | sb |  |  |  |  | 2 |  |  |  |  |  |  | 2 | 2 | 0.06 |
| *Noterus clavicornis* | Not cla | sb | 11 |  |  |  | 2 | 1 |  |  |  |  | 11 | 3 | 14 | 0.43 |
| *Noterus crassicornis* | Not cra | sb | 12 |  | 1 |  | 14 | 1 |  |  |  |  | 7 | 21 | 28 | 0.86 |
| *Agabus biguttatus* | Aga big | kr |  | 4 |  |  |  |  |  |  |  |  | 1 | 3 | 4 | 0.12 |
| *Agabus bipustulatus* | Aga bip | sb | 249 | 2 | 4 | 19 | 1 | 3 |  |  |  |  | 143 | 135 | 278 | 8.51 |
| *Agabus chalconatus* | Aga chal | sb | 2 |  |  |  |  |  |  |  |  |  |  | 2 | 2 | 0.06 |
| *Agabus didymus* | Aga did | kr |  |  |  | 1 | 1 |  |  |  |  |  | 2 |  | 2 | 0.06 |
| *Agabus paludosus* | Aga pal | re | 19 |  |  | 1 | 1 |  |  |  |  |  | 6 | 15 | 21 | 0.64 |
| *Agabus* sp. | Aga spe | sb | 25 | 2 | 14 | 1 | 6 |  |  |  |  |  | 20 | 28 | 48 | 1.47 |
| *Agabus sturmii* | Aga stu | sb | 13 |  |  |  |  |  |  |  |  |  | 2 | 11 | 13 | 0.40 |
| *Agabus uliginosus* | Aga uli | sb | 2 |  |  |  |  |  |  |  |  |  | 2 |  | 2 | 0.06 |
| *Agabus undulatus* | Aga und | sb | 4 | 1 |  |  |  |  |  |  |  |  | 4 | 1 | 5 | 0.15 |
| *Ilybius ater* | Ily ate | sa |  |  |  | 6 |  |  |  |  |  |  | 2 | 4 | 6 | 0.18 |
| *Ilybius fenestratus* | Ily fen | re | 4 |  | 2 | 2 |  |  |  |  |  |  | 4 | 4 | 8 | 0.24 |
| *Ilybius fuliginosus* | Ily ful | re | 58 | 11 | 23 | 43 | 31 | 1 |  | 2 |  |  | 51 | 118 | 169 | 5.17 |
| *Ilybius quadriguttatus* | Ily qua | sa | 1 |  | 1 | 9 | 2 | 1 |  |  |  |  | 2 | 12 | 14 | 0.43 |
| *Ilybius similis* | Ily sim | sa |  |  |  | 3 |  |  |  |  |  |  | 3 |  | 3 | 0.09 |
| *Ilybius* sp. | Ily spe | sa | 5 |  |  |  |  |  |  |  |  |  | 4 | 1 | 5 | 0.15 |
| *Ilybius subaeneus* | Ily sub | sa |  |  | 1 |  |  |  |  |  |  |  |  | 1 | 1 | 0.03 |
| *Platambus maculatus* | Pla mac | re |  |  |  | 2 | 1 |  |  |  |  |  | 2 | 1 | 3 | 0.09 |
| *Colymbetes fuscus* | Col fus | sb | 1 |  |  | 3 | 14 | 45 |  |  |  |  | 44 | 19 | 63 | 1.93 |
| *Colymbetes paykulli* | Col pay | sa |  |  |  | 5 |  | 2 |  |  |  |  | 5 | 2 | 7 | 0.21 |
| *Colymbetes striatus* | Col str | sa | 7 |  |  | 5 | 13 | 4 |  |  |  |  | 12 | 17 | 29 | 0.89 |
| *Rhantus exsoletus* | Rha exo | sb |  |  |  |  |  | 6 |  |  |  |  |  | 6 | 6 | 0.18 |
| *Rhantus frontalis* | Rha fro | sb | 3 |  |  |  |  | 1 |  |  |  |  | 2 | 2 | 4 | 0.12 |
| *Rhantus incognitus* | Rha inc | re |  |  |  |  | 1 |  |  |  |  |  |  | 1 | 1 | 0.03 |
| *Rhantus latitans* | Rha lat | sb |  |  |  |  | 2 |  |  |  |  |  |  | 2 | 2 | 0.06 |
| *Rhantus* sp. | Rha spe | sb |  | 2 |  | 2 |  |  |  |  |  |  | 1 | 3 | 4 | 0.12 |
| *Rhantus suturalis* | Rha sut | sb |  |  |  | 7 | 9 | 1 |  |  |  |  | 10 | 7 | 17 | 0.52 |
| *Acilius canaliculatus* | Aci can | sa |  |  |  | 17 | 27 | 1 | 1 | 1 |  |  | 23 | 24 | 47 | 1.44 |
| *Acilius sulcatus* | Aci can | sb |  |  |  | 3 | 8 | 2 |  |  |  |  | 2 | 11 | 13 | 0.40 |
| *Graphoderus austriacus* | Gra aus | sb |  |  |  |  | 1 |  |  |  |  |  | 1 |  | 1 | 0.03 |
| *Graphoderus cinereus* | Gra cin | sb | 1 |  |  |  |  |  |  |  |  |  |  | 1 | 1 | 0.03 |
| *Cybister lateralimarginalis* | Cyb lat | sb | 1 |  |  |  |  | 1 |  |  |  |  | 1 | 1 | 2 | 0.06 |
| *Dytiscus circumcintus* | Dyt cic | sb |  |  |  |  | 1 |  |  |  |  |  |  | 1 | 1 | 0.03 |
| *Dytiscus circumflexus* | Dyt cif | sb |  |  |  |  | 1 |  |  |  |  |  |  | 1 | 1 | 0.03 |
| *Dytiscus dimidiatus* | Dyt dim | sb | 2 |  | 1 | 2 | 3 | 12 |  |  | 2 |  | 8 | 14 | 22 | 0.67 |
| *Dytiscus marginalis* | Dyt mar | sb | 2 |  | 1 | 3 | 4 | 2 |  | 1 |  |  | 6 | 7 | 13 | 0.40 |
| *Dytiscus* sp. | Dyt spe | sb | 4 |  |  |  |  |  |  |  |  |  | 2 | 2 | 4 | 0.12 |
| *Hydaticus continentalis* | Hyd con | sb |  |  |  |  | 1 |  |  |  |  |  |  | 1 | 1 | 0.03 |
| *Hydaticus seminiger* | Hyd sem | sb | 4 |  |  | 5 | 6 | 2 |  |  |  |  | 7 | 10 | 17 | 0.52 |
| *Hydaticus transversalis* | Hyd tra | sb |  | 1 |  |  |  | 7 |  |  |  |  |  | 8 | 8 | 0.24 |
| *Hydroglyphus geminus* | Hyd gem | sa |  |  |  |  | 1 |  |  |  |  |  |  | 1 | 1 | 0.03 |
| *Graptodytes granularis* | Gra gra | sb | 1 |  |  |  | 1 |  |  |  |  |  |  | 2 | 2 | 0.06 |
| *Graptodytes pictus* | Gra pic | sb |  |  |  |  |  | 1 |  |  |  |  |  | 1 | 1 | 0.03 |
| *Hydroporus angustatus* | Hyd ang | sa | 11 |  |  | 3 |  |  |  |  |  |  | 2 | 12 | 14 | 0.43 |
| *Hydroporus erytrocephalus* | Hyd ery | sa |  | 1 |  | 2 |  |  |  | 2 |  |  |  | 5 | 5 | 0.15 |
| *Hydroporus incognitus* | Hyd inc | sa | 5 |  |  | 20 | 12 | 14 |  |  | 1 |  | 14 | 38 | 52 | 1.59 |
| *Hydroporus nigrita* | Hyd nig | sa |  |  |  |  |  |  |  | 2 |  |  | 2 |  | 2 | 0.06 |
| *Hydroporus obscurus* | Hyd obs | sa |  |  | 5 | 6 |  |  |  |  |  |  | 5 | 6 | 11 | 0.34 |
| *Hydroporus palustris* | Hyd pal | sb | 23 |  |  | 2 | 10 | 7 |  |  |  |  | 7 | 35 | 42 | 1.29 |
| *Hydroporus planus* | Hyd pla | sa | 5 |  |  |  |  |  |  |  |  |  | 3 | 2 | 5 | 0.15 |
| *Hydroporus rufifrons* | Hyd ruf | sa |  | 2 |  |  |  |  |  |  |  |  |  | 2 | 2 | 0.06 |
| *Hydroporus scalesianus* | Hyd sca | sa |  |  |  | 1 |  |  |  |  |  |  | 1 |  | 1 | 0.03 |
| *Nebrioporus canaliculatus* | Neb can | sa |  |  |  |  | 1 |  |  |  |  |  |  | 1 | 1 | 0.03 |
| *Porhydrus lineatus* | Por lin | sa | 1 |  |  | 2 |  |  |  |  |  |  | 2 | 1 | 3 | 0.09 |
| *Suphrodytes dorsalis* | Sup dor | sb |  |  |  |  | 1 |  |  |  |  |  | 1 |  | 1 | 0.03 |
| *Suphrodytes figuratus* | Sub fig | sb |  |  |  | 1 |  |  |  |  |  |  | 1 |  | 1 | 0.03 |
| *Hygrotus decoratus* | Hyg dec | sa | 4 | 1 |  | 4 | 1 |  |  |  | 1 |  | 4 | 7 | 11 | 0.34 |
| *Hygrotus impressopunctatus* | Hyg imp | sb | 2 |  |  | 1 | 1 | 2 |  |  |  |  | 3 | 3 | 6 | 0.18 |
| *Hygrotus inaequalis* | Hyg ine | sb | 8 |  |  | 13 | 7 |  |  |  |  |  | 11 | 17 | 28 | 0.86 |
| *Hygrotus novemlineatus* | Hyg nov | sa |  |  |  |  | 1 |  |  |  |  |  |  | 1 | 1 | 0.03 |
| *Hyphydrus ovatus* | Hyp ova | sb | 1 |  | 1 | 26 | 34 | 2 |  |  |  |  | 21 | 43 | 64 | 1.96 |
| *Laccophilus hyalinus* | Lac hal | re |  |  |  | 4 | 11 | 2 |  |  | 1 |  | 7 | 11 | 18 | 0.55 |
| *Laccophilus minutus* | Lac min | sb | 17 |  |  |  |  |  |  |  |  |  | 5 | 12 | 17 | 0.52 |
| *Laccophilus* sp. |  | sb | 1 |  |  |  |  |  |  |  |  |  |  | 1 | 1 | 0.03 |
| *Helophorus aquaticus* | Hel aqu | sb | 1 |  |  |  |  | 1 |  |  |  |  |  | 2 | 2 | 0.06 |
| *Helophorus flavipes* | Hel fla | sa | 9 |  |  |  |  |  |  |  |  |  | 1 | 8 | 9 | 0.28 |
| *Helophorus granularis* | Hel gra | sa | 1 |  | 2 | 19 | 1 | 1 |  | 1 | 1 |  | 18 | 8 | 26 | 0.80 |
| *Helophorus griseus* | Hel gri | sa | 10 | 2 | 6 | 2 |  | 2 |  | 7 |  |  | 14 | 15 | 29 | 0.89 |
| *Helophorus minutus* | Hel min | sa | 1 |  |  | 1 | 1 |  |  |  |  |  | 2 | 1 | 3 | 0.09 |
| *Helophorus nanus* | Hel nan | sa | 1 |  |  |  |  |  |  |  |  |  |  | 1 | 1 | 0.03 |
| *Helophorus nubilus* | Hel nub | sb |  |  |  |  |  |  |  | 1 |  |  |  | 1 | 1 | 0.03 |
| *Helophorus strigifrons* | Hel str | sa | 2 |  |  |  |  |  |  |  |  |  | 2 |  | 2 | 0.06 |
| *Hydrochus angustatus* | Hyd ang | sb |  |  | 1 |  |  |  |  |  |  |  |  | 1 | 1 | 0.03 |
| *Hydrochus brevis* | Hyd bre | sb | 1 |  |  | 1 | 1 |  |  |  |  |  | 1 | 2 | 3 | 0.09 |
| *Hydrochus elongatus* | Hyd elo | sb | 7 |  |  |  |  |  |  |  |  |  | 3 | 4 | 7 | 0.21 |
| *Hydrochus ignicollis* | Hyd ign | sa |  |  |  |  | 1 |  |  |  |  |  |  | 1 | 1 | 0.03 |
| *Anacaena globulus* | Ana glo | sb | 14 |  |  |  |  |  |  |  |  |  | 4 | 10 | 14 | 0.43 |
| *Anacaena limbata* | Ana lim | sb | 18 | 7 | 33 | 6 | 8 | 1 |  | 3 | 3 |  | 11 | 68 | 79 | 2.42 |
| *Anacaena lutescens* | Ana lut | sa | 35 | 2 | 6 | 1 | 1 |  |  |  | 1 |  | 9 | 37 | 46 | 1.41 |
| *Cymbiodyta marginella* | Cym mar | sa | 7 |  |  |  |  |  |  |  |  |  | 6 | 1 | 7 | 0.21 |
| *Enochrus coarctatus* | Eno coa | sa |  |  |  |  | 1 | 1 |  |  |  |  |  | 2 | 2 | 0.06 |
| *Enochrus melanocephalus* | Eno mal | sa | 1 |  | 1 |  | 5 |  |  | 1 |  |  | 5 | 3 | 8 | 0.24 |
| *Enochrus quadripunctatus* | Eno qua | sb | 1 |  |  | 1 |  |  |  |  |  |  | 1 | 1 | 2 | 0.06 |
| *Enochrus testaceus* | Eno tes | sb |  |  |  | 2 |  |  |  | 1 |  |  | 3 |  | 3 | 0.09 |
| *Helochares obscurus* | Helobs | sb | 4 |  |  | 4 |  |  |  | 1 |  |  | 7 | 2 | 9 | 0.28 |
| *Hydrobius fuscipes* | Hyd fus | sb | 42 |  | 8 | 1 | 7 | 1 |  | 2 |  |  | 23 | 38 | 61 | 1.87 |
| *Hydrochara caraboides* | Hyd car | sb | 2 |  |  | 10 | 1 | 1 |  | 3 |  |  | 13 | 4 | 17 | 0.52 |
| *Laccobius bipunctatus* | Lcc bip | sa | 2 |  |  |  |  |  |  |  |  |  | 2 |  | 2 | 0.06 |
| *Laccobius minutus* | Lcc min | sa | 36 |  | 52 |  | 1 |  |  |  |  |  | 15 | 74 | 89 | 2.72 |
| *Coelostoma orbiculare* | Coe orb | sb | 1 |  | 1 |  |  |  |  |  |  |  | 1 | 1 | 2 | 0.06 |
| *Cercyon bifenestratus* | Cer bif | sb |  |  | 1 |  | 1 | 1 |  |  |  |  |  | 3 | 3 | 0.09 |
| *Cercyon convexiusculus* | Cer con | sb | 1 |  |  |  | 2 |  |  |  |  |  | 3 |  | 3 | 0.09 |
| *Cercyon tristis* | Cen tri | sb | 2 |  | 1 |  |  |  |  |  | 1 |  | 2 | 2 | 4 | 0.12 |
| *Cryptopleurum minutum* | Cry min | sb | 2 |  |  |  |  |  |  |  |  |  | 2 |  | 2 | 0.06 |
| *Hydraena bitteni* | Hdr bri | sb | 1 |  |  |  |  |  |  |  |  |  |  | 1 | 1 | 0.03 |
| *Hydraena gracilis* | Hdr gra | rb |  |  |  |  |  |  |  |  | 1 |  | 1 |  | 1 | 0.03 |
| *Hydraena palustris* | Hdr pls | sa | 10 | 464 | 17 | 105 | 91 |  | 24 | 109 | 271 |  | 939 | 152 | 1091 | 33.38 |
| *Hydraena riparia* | Hdr rip | rb | 19 |  |  |  |  | 7 |  |  |  | 19 | 33 | 12 | 45 | 1.38 |
| *Limnebius aluta* | Lim alu | sb | 1 |  |  |  |  |  |  |  |  |  | 1 |  | 1 | 0.03 |
| *Limnebius atomus* | Lim ato | sb | 4 |  | 2 | 1 |  |  |  | 1 |  |  | 3 | 5 | 4 | 0.24 |
| *Limnebius crinifer* | Lim cri | sb | 1 |  |  |  |  |  |  |  |  |  | 1 |  | 1 | 0.03 |
| *Limnebius parvulus* | Lim par | sb | 13 | 5 | 1 | 10 | 3 |  |  | 3 | 4 |  | 17 | 22 | 39 | 1.20 |
| *Limnebius truncatellus* | Lim trun | rb |  |  |  | 2 |  |  |  | 4 |  |  | 4 | 2 | 6 | 0.18 |
| *Ochthebius minimus* | Och min | sb | 5 |  | 1 |  | 4 | 1 |  | 8 | 4 |  | 8 | 15 | 23 | 0.70 |
| *Elmis aenea* | Elm aen | rb |  |  |  |  |  |  |  | 3 | 23 |  | 26 |  | 26 | 0.80 |
| *Elmis maugetii* | Elm mau | rb |  |  |  |  |  |  |  |  |  | 3 | 3 |  | 3 | 0.09 |
| *Limnius volckmari* | Lim vol | rb |  |  |  |  |  |  |  | 1 | 1 |  | 2 |  | 2 | 0.06 |
| *Oulimnius tuberculatus* | Oul tub | rb |  | 1 |  |  | 6 |  |  | 10 | 3 | 6 | 23 | 3 | 26 | 0.80 |
| *Elodes* sp. | Elo sp. | re | 180 |  |  |  |  |  |  |  |  |  | 144 | 36 | 180 | 5.51 |
| *Prionocyphon serricornis* | Pri ser | sb | 3 |  | 2 |  | 1 |  |  |  |  |  |  | 6 | 6 | 0.03 |
| *Scirtes sp.* | Sci sp | sb | 1 | 25 | 1 |  | 2 |  |  | 3 | 2 |  | 8 | 26 | 34 | 1.04 |
| *Tanysphyrus lemnae* | Tan lem | sb |  |  |  |  | 3 |  |  |  |  |  |  | 3 | 3 | 0.09 |
| *Donacia sparganii* | Don lac | sb |  |  |  |  | 1 |  |  |  |  |  | 1 |  | 1 | 0.03 |
| *Georissus* sp. | Geo sp. | sb |  | 1 |  |  |  |  |  | 1 |  |  |  | 2 | 2 | 0.06 |
| *Heterocerus* sp. | Het sp. | sb |  |  |  | 1 |  |  |  |  |  |  | 1 |  | 1 | 0.03 |
