# Supplementary material for: Sequentiality of beetle communities in the longitudinal gradient of a lowland river in the context of the river continuum concept
Source: PeerJ. 2022 Apr 5;10:e13232. doi: 10.7717/peerj.13232 (PMC8992647; doi:10.7717/peerj.13232)
Supplement: Supplemental Information 2 — Column. coordinates and contributions to Inertia. CN, Column number. Coordin, Coordinates. Dim, Dimension. [file peerj-10-13232-s002.docx]

Appendix 2. Results of Multidimensional Correspondence Analysis (MCA) showing the relationships between ecological groups of beetle communities (re, rb, sa, sb, kr), kind of a section of the river course (upper, middle, lower) and different mesohabitats (current and pocket river) Column coordinates and contributions to Inertia. CN – Column number. Coordin – Coordinates. Dim – Dimension.

| **Variables (name column)** | CN | Coordin  Dim 1 | Coordin  Dim 2 | Mass | Quality | Relative  Inertia | **Inertia**  **Dim 1** | Cosine ^2^  Dim 1 | **Inertia**  **Dim 2** | Cosine ^2^  Dim 1 |
| --- | --- | --- | --- | --- | --- | --- | --- | --- | --- | --- |
| **Ecological groups** | | | | | | | | | |  |
| Reophiles –r | 1 | -0.47613 | -1.1939 | 0.04673 | 0.26939 | 0.12283 | 0.01978 | 0.03696 | 0.17174 | 0.23243 |
| Stagnobionts a –sa | 2 | 0.59160 | -0.0953 | 0.15656 | 0.31803 | 0.07575 | 0.10231 | 0.30999 | 0.00366 | 0.00804 |
| Stagnobionts b –sb | 3 | -0.79149 | 0.3610 | 0.11822 | 0.41597 | 0.09218 | 0.13828 | 0.34431 | 0.03973 | 0.07165 |
| Reobionts – rb | 4 | 2.10618 | 2.5685 | 0.01119 | 0.38327 | 0.13806 | 0.09268 | 0.15409 | 0.19033 | 0.22917 |
| Krenobionty – kr | 5 | -0.59092 | -1.1451 | 0.00062 | 0.00310 | 0.14259 | 0.00040 | 0.00065 | 0.00210 | 0.00245 |
| **Section of the river course** | | | | | | | | | | |
| upper course | 6 | -0.18104 | -0.5390 | 0.220184 | 0.629301 | 0.048492 | 0.013475 | 0.063782 | 0.164968 | 0.565519 |
| middle course | 7 | -0.46744 | 1.2151 | 0.076469 | 0.504661 | 0.110085 | 0.031197 | 0.065049 | 0.291123 | 0.439612 |
| lower course | 8 | 2.06127 | 0.7026 | 0.036680 | 0.586401 | 0.127137 | 0.290984 | 0.525355 | 0.046689 | 0.061047 |
| **mesohabitats** | | | | | | | | | | |
| pocket river | 9 | -0.84830 | 0.3876 | 0.136566 | 0.603813 | 0.084329 | 0.183510 | 0.499506 | 0.052914 | 0.104307 |
| river current | 10 | 0.58879 | -0.2690 | 0.196767 | 0.603813 | 0.058528 | 0.127365 | 0.499506 | 0.036725 | 0.104307 |
| **sites** | | | | | | | | | | |
| K1 | 11 | -0.43580 | -0.5224 | 0.34431 | 0.18565 | 0.07575 | 0.10231 | 0.07617 | 0.03173 | 0.10947 |
| K2 | 12 | 0.28829 | -0.6215 | 0.15409 | 0.10238 | 0.09218 | 0.13828 | 0.01812 | 0.07587 | 0.08425 |
| K3 | 13 | -0.65340 | -0.1593 | 0.00065 | 0.02792 | 0.13806 | 0.09268 | 0.02635 | 0.38760 | 0.00156 |
| K6 | 14 | -0.06166 | -0.6272 | 0.34431 | 0.06310 | 0.110085 | 0.031197 | 0.00060 | 0.13828 | 0.06249 |
| K8 | 15 | -0.67768 | 1.1793 | 0.15409 | 0.26494 | 0.127137 | 0.290984 | 0.06577 | 0.09268 | 0.19917 |
| K9 | 16 | -0.67512 | 1.3935 | 0.00065 | 0.10665 | 0.04673 | 0.26939 | 0.02027 | 0.13167 | 0.08638 |
| K10 | 17 | -0.48381 | 1.3106 | 0.34431 | 0.01590 | 0.15656 | 0.31803 | 0.00190 | 0.06189 | 0.01399 |
| K11 | 18 | 0.19300 | 1.1428 | 0.15409 | 0.07587 | 0.11822 | 0.41597 | 0.00210 | 0.127137 | 0.07377 |
| K12 | 19 | 1.984070 | 0.5414 | 0.00065 | 0.47695 | 0.110085 | 0.031197 | 0.44389 | 0.031197 | 0.03305 |
| K14 | 20 | 2.96012 | 2.5799 | 0.10238 | 0.13537 | 0.127137 | 0.290984 | 0.07693 | 0.041167 | 0.05844 |
